# Harnessing the Power from Ambient Moisture with Hygroscopic Materials

**DOI:** 10.1007/s40820-025-01983-y

**Published:** 2026-01-05

**Authors:** Daozhi Shen, Fangzhou Li, Yanjie Su, Limin Zhu

**Affiliations:** 1 School of Mechanical Engineering, Shanghai Jiao Tong University, Shanghai, 200240 People’s Republic of China; 2 National Center for Translational Medicine, Shanghai Jiao Tong University, Shanghai, 200240 People’s Republic of China; 3 Department of Micro/Nano Electronics, School of Electronics Information and Electrical Engineering, Shanghai Jiao Tong University, Shanghai, 200240 People’s Republic of China

**Keywords:** Moisture electricity generation, Hydroelectricity, Nanogenerators, Materials design, Hygroscopic material

## Abstract

Moisture electricity generation (MEG) has emerged as a sustainable and versatile energy-harvesting technology capable of converting ubiquitous environmental moisture into electrical energy, which holds great promise for renewable energy and constructing self-powered electronics. In this review, we begin by outlining the fundamental mechanisms—ion diffusion, electric double layer formation, and streaming potential—that govern charge transport for MEG in moist environments. A comprehensive survey of material innovations follows, highlighting breakthroughs in carbon-based materials, conductive polymers, hydrogels, and bio-inspired systems that enhance MEG performance, scalability, and biocompatibility. We then explore a range of device architectures, from planar and layered systems to flexible, miniaturized, and textile-integrated designs, engineered for both energy conversion and sensor integration. Key challenges are analyzed, along with strategies for overcoming them. We conclude with a forward-looking perspective on future directions, including hybrid energy systems, AI-assisted material design, and real-world deployment. This review presents a timely and comprehensive overview of MEG technologies and their trajectory toward practical and sustainable energy solutions.

## Highlights

- Typical structures/working mechanisms of moisture electricity generation (MEG) devices are comprehensively reviewed.
- An extensive comparison of the power generation between various materials and architectures are summarized.
- Applications, challenges, and future development directions of MEG technology, especially the artificial intelligence-assisted material discovery, are discussed.

## Graphical abstract

**Figure:**
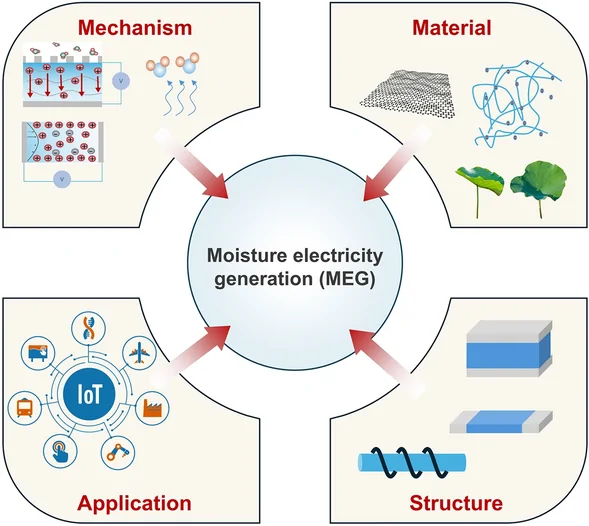

## Introduction

Moisture in the atmosphere is considered as an abundant and renewable source of low-grade energy, which has recently become the focus of new energy-harvesting technologies [1–7]. While water has long been used for energy generation through large-scale systems, converting gaseous water into electricity at the nanoscale is a much newer concept. This method, often referred to as moisture electricity generation (MEG), takes advantage of natural interaction between functional materials with water vapor to generate electricity due to unbalanced ion movement inside without complex mechanical parts or external fuels [8–11]. Since the first demonstrations, moisture electricity generation technique has gained a great attention, showing strong potential for clean and distributed energy generation from the air on earth [12–19].

A major breakthrough came in 2015, when Qu and colleagues discovered that a film of graphene oxide (GO) could produce continuous voltage when exposed to a moisture environment [20]. This effect was coming from the unbalanced distribution of oxygen-based groups in the GO, which caused directional ion flow once water was absorbed to release protons. Thereafter, similar results using other type nanomaterials such as carbon black and nanowires have been demonstrated [21–23]. In the following years, the performance of such devices improved rapidly. A 3D GO structure with abundant porous structures was built in 2016 to enhance ion transport and increase output due to the high density of diffusion channels [24]. By 2018, graphene-based films reached output voltages of nearly 1.5 V by modifying interfacial properties with electrodes [25]. In addition, porous carbon materials also demonstrated reliable voltage generation (~ 1 V) due to evaporation-driven water flow [26]. These developments confirmed that when a moisture gradient is maintained across a material, ion movement can be harnessed to produce electricity.

Moisture-electric generation works by converting the interaction between water molecules and a material into electric energy, typically involving water and corresponding ions diffusing through an active material and creating a charge difference that results in electrical output [27–29]. Several physical mechanisms have been identified, although there are still some debates on them [6, 30–32]. One is the ion-gradient mechanism, which relies on directional migration of ions released from the functional groups during the interaction with water molecules due to the water or functions groups gradient inside. These materials include GO [22, 25, 33], polymers [15, 34, 35], and biopolymers [27, 36, 37] that have hydrophilic –COOH functional groups that can highly absorb water. Another mechanism is known as the streaming potential, which involves the formation of electric double layers (EDLs). When water is absorbed, charged surfaces in the material can attract counter-ions, creating a separation of charge across regions if there is a gradient in moisture or surface properties. The ions then migrate with the moving of water and thus producing electricity [9, 26, 38, 39]. In some cases, the two mechanisms may happen together, and the exact cause of electricity in a specific device may be different [40].

**Fig. 1:**
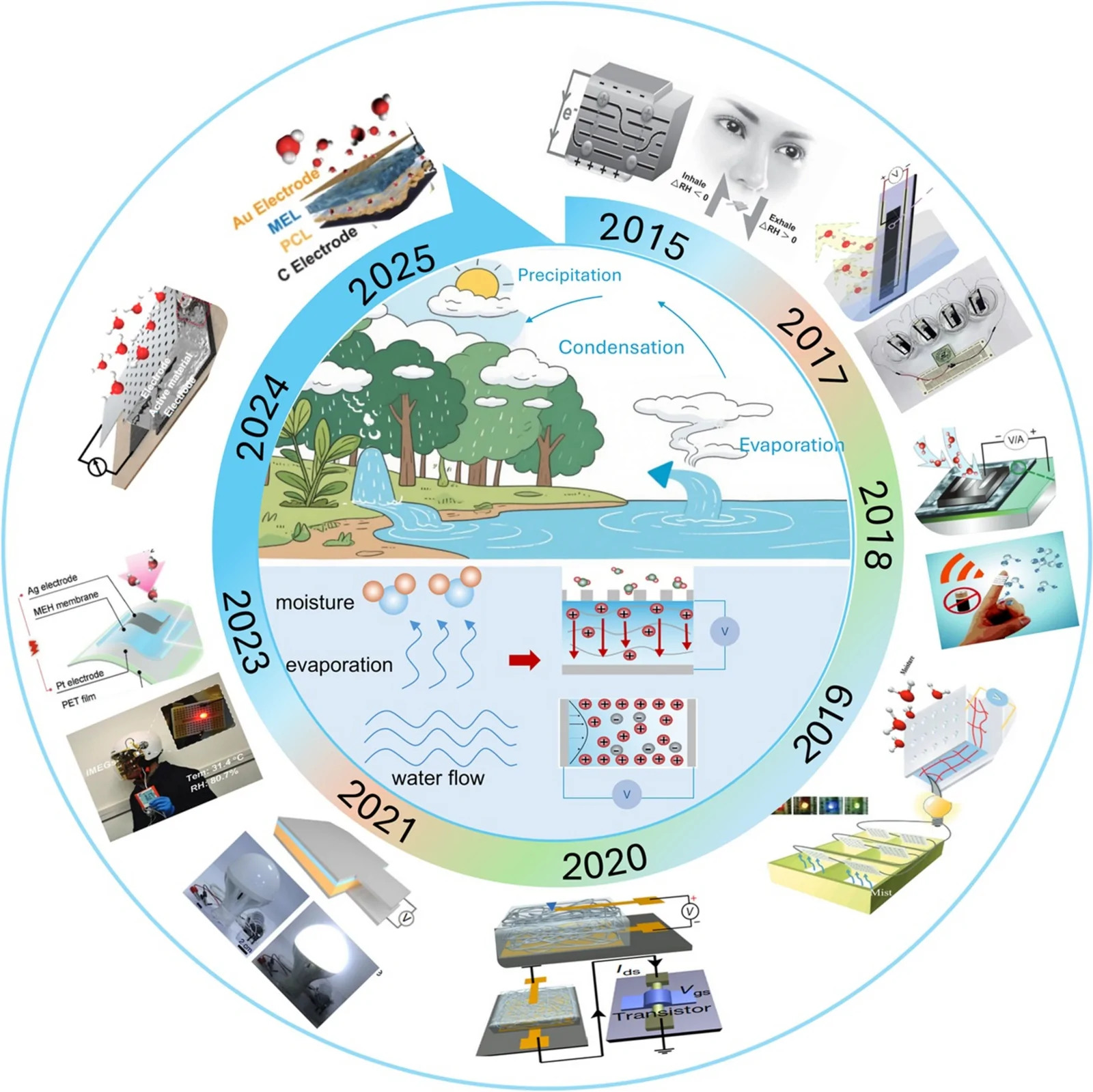
Summary of the development of MEG technology [15, 20, 26, 27, 29, 34, 47, 49, 65]

The materials used for MEGs have been developed significantly since the first GO devices. Among them, carbon-based materials have been widely used for MEGs, including reduced graphene oxide, carbon nanotubes, and porous carbon films [40–44]. For example, a GO film that could generate about 0.3 V in ambient air due to internal moisture gradients [24]. The printed porous carbon films that created electricity from water flow within nanochannels [26]. Polymers, especially hydrophilic ones, have also become key materials for MEGs. Some polymers can absorb large amounts of water and release ions, which can facilitate the electrical output [45, 46]. A sulfonated polyether ketone membrane can boost the electricity output by improving moisture capture and ion exchange (Fig. 1) [47]. An important polymer for MEGs is hydrogel-based material. They have water-rich polymer networks and are particularly promising as they can combine moisture retention with high ionic conductivity [48]. A hydrogel from PVA and alginate that produced ~ 1.3 V and current densities exceeding 1000 µA cm^−2^ under ambient conditions [49]. Doping hydrogels with salts such as LiCl or adding biopolymers further improves performance. For example, a hydrogel using sulfated cellulose nanofibers that operated continuously for over 600 h [29]. In addition to polymers, there has also been increasing interest in sustainable and biodegradable materials for MEGs. The biomaterial based on protein nanowires from the bacterium Geobacter to generate 0.5 V under ambient conditions [27]. Other biological materials, including cellulose and even DNA, have also been used for harvesting moisture energy from air, shown to be recyclable and environmental friendly [13, 50].

Device architecture plays a big role in MEG performance. In-plane designs maintain a horizontal moisture gradient and can run for many hours as long as the water is provided [26]. The vertical or sandwiched devices, with water concentration difference across thickness, typically produce higher currents but run for shorter periods due to faster moisture equilibration [20]. Asymmetric designs have been introduced to solve this problem. The composite films with different porosities of released ions on two side [15, 51–53] or layered materials with different water-absorbing properties [54–56] can improve the electricity output and maintain internal gradients much longer. Some designs use moisture buffer layers such as hydrogels to feed vapor to one side continuously [54, 57–59]. In addition to the single MEG devices design, larger systems have been created by connecting MEGs in series or parallel to improve the overall voltage or current, respectively. For instance, an array of 160 MEG units produced over 150 V, and a nearly 2 mA of current can be obtained by parallel integration [60]. Early MEG devices produced only millivolts and nanoamp currents [20, 26], but now single-layer devices can exceed 1 V and reach current densities of hundreds of μA cm^−2^ [49]. Power density has also increased from nanowatt levels to as high as 10–3 W cm^−2^. Importantly, devices with internal buffers and regenerating gradients can now operate continuously for days or even weeks or longer [55, 61]. Despite these advances, MEG efficiency is still low, typically less than 5% of the theoretical efficiency from water adsorption [5, 62]. Improving materials, reducing charge recombination, and maintaining moisture gradients are critical areas of ongoing research [2, 7, 8, 63, 64].

In this review, we begin by outlining the fundamental mechanisms—ion diffusion and streaming potential—that govern charge transport for MEG in moist environments. A comprehensive survey of material innovations follows, highlighting breakthroughs in carbon-based materials, conductive polymers, hydrogels, and bio-inspired systems that enhance MEG performance, scalability, and biocompatibility. We then explore a range of device architectures, from planar and layered systems to flexible, miniaturized, and textile-integrated designs, engineered for both energy conversion and sensor integration. Key challenges are analyzed, along with strategies for overcoming them. We conclude with a forward-looking perspective on future directions, including hybrid energy systems, AI-assisted material design, and real-world deployment. This review presents a timely and comprehensive overview of MEG technologies and their trajectory toward practical and sustainable energy solutions.

## Fundamental Mechanisms

Ambient moisture can be converted into electrical energy through several fundamental interfacial mechanisms, primarily by ion diffusion and streaming potentials based on EDL effects that occur spontaneously at water–solid interfaces [66–69]. These processes do not require thermal gradients, mechanical motion, or chemical fuel, making moisture-enabled electricity generation a unique technique for harvesting low-grade energy in ambient environment [2, 4, 70–72]. In this section, we provide an in-depth discussion of the physics and chemistry underlying each mechanism and how they contribute to moisture-electricity generation. The brief comparison between the two mechanisms is summarized in Table 1.

**Table 1:** Comparison between ion diffusion and streaming potential mechanisms

Mechanism	Material requirement	Driving force	Main device architecture
Ion diffusion	Charged micro/nano channels	Water evaporation	Planar
Streaming potential	Functional groups	Water absorption	Sandwiched

### Ion Diffusion Mechanism

**Fig. 2:**
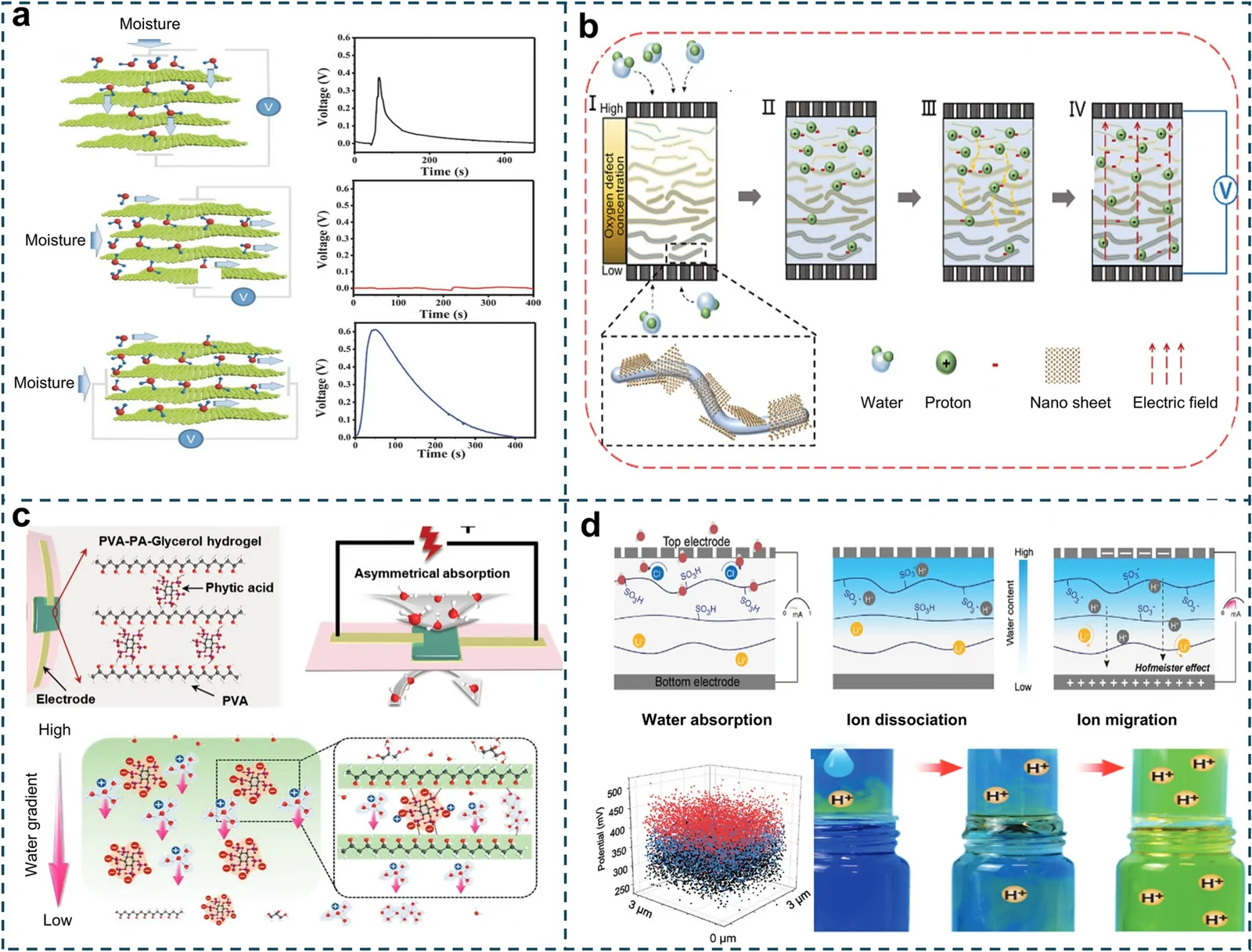
Moisture electricity generation based on ion diffusion mechanism. **a** Schematic of the ion diffusion from GO film. Reproduced with permission [22]. Copyright 2018, Wiley–VCH. **b** Charge migration driven by the concentration difference. Reproduced with permission [73]. Copyright 2024, Wiley–VCH. **c** Prolonged generation from a sustained gradient from an ionic hydrogel engineered through the molecular modification. **d** Ion mobility modification to improve the output. Reproduced with permission [34]. Copyright 2023, Wiley–VCH

The ion diffusion mechanism is also called the concentration-gradient or gradient diffusion mechanism, which is based on the creation of ionic concentration gradients across a moisture-absorbing material, leading to charge separation and thus give electric output. Many hygroscopic solids contain surface functional groups, including –OH, –COOH, and –SO_3_H, that dissociate and release mobile ions (such as protons) upon absorbing water molecules [5, 10, 12]. When there is an asymmetry presented in the material chemical composition or the moisture distribution, a concentration gradient of these ions can be established. The mobile ions will diffuse from the region of higher concentration (e.g., a higher humidity or chemically richer region) to a region of lower concentration (Fig. 2a) [22]. This ion migration under a gradient effectively converts the chemical potential energy of adsorbed water into an electric potential difference due to the ion concentration difference across the functional materials. Here, the moisture gradient or functional-group gradient acts as the driving force for ions diffusion, and the charge separation produces a built-in electric field and measurable voltage (Fig. 2b) [73]. In some cases, the ion diffusion can also be driven by the difference in ion hydrations. For instance, an ion with a stronger hydration might move more slowly or differently compared to an ion with weaker hydration. When a moisture gradient is present, these differences can cause a selective movement of ions, leading to charge separation and a voltage output [40].

If the moisture gradient is transient during the interaction, the device may produce a pulse of current that decays over time as concentrations equalize. However, the output from MEGs often keeps existing as long as a moisture gradient is introduced and can be maintained until ionic equilibrium is reached [61]. In some designs, by keeping one side of a film in a humid environment and the other side in dry, a sustained gradient can be formed, which allows for continuous or prolonged generation (Fig. 2c) [16]. Generally, ion-diffusion-based MEGs produce an open-circuit voltage on the order of tens to hundreds of millivolts. In recent years, though engineered methods such as asymmetric structuring designing and multi-device stacking, the voltages have been significantly improved. Meanwhile, the internal resistance and output current depend on ionic mobility and material conductivity [74]. The currents are often in the nanoamp to microamp range for single devices, but similarly, they can be increased through modifying ionic mobility and density of active materials such as hydrogels or by parallel integration (Fig. 2d) [34].

The ion diffusion mechanism can be described by coupled ion transport and electrostatic equations. The Nernst–Planck equation for ion diffusion and migration together with Poisson’s equation for the electric field form the basis for theoretical modeling. This can be simplified as a one-dimensional diffusion cell, with one end of the material has a higher ionic chemical potential than the other, leading to a Nernst-like potential difference. In practice, fully quantitative prediction is complex because the ion generation is coupled to water adsorption thermodynamics and dissociation equilibria of surface groups. Overall, the ion diffusion mechanism is relatively well-established as a primary driver in many moisture-electric generators, especially those using hygroscopic functional polymers, graphene oxide materials, or biopolymers. It provides a straightforward route to harvest ambient humidity by leveraging chemical potentials. The ongoing research is focused on enhancing the ion generation through chemical design and maintaining sharper gradients through structural or environmental control to improve both voltage and current outputs.

To confirm the ion diffusion mechanism, a variety of experiments have been carried out to investigate the dynamics of ions during the interaction of active materials with water. For instance, Kelvin probe force microscopy (KPFM) and pH indicator testing provide microscopic evidence of ion migration and accumulation [34]. It is shown that when one side of a sulfonated polyelectrolyte membrane was exposed to moisture, protons accumulated on the other side, creating a measurable surface potential difference through KPFM. This confirms that protons released by dissociation migrate across the material from the wet to the dry side to form a charge difference. As the protons accumulation also change the pH locally, a pH indicator is a cost-effective way to characterize the charges diffusion with water [47]. As the region of higher humidity releases more ions, and the resultant ion diffusion toward the drier side drives a current [17, 45, 46, 48, 75–78]. Such a configuration essentially creates a continuous chemical potential as long as the humidity gradient is maintained.

### Streaming Potential Mechanism

**Fig. 3:**
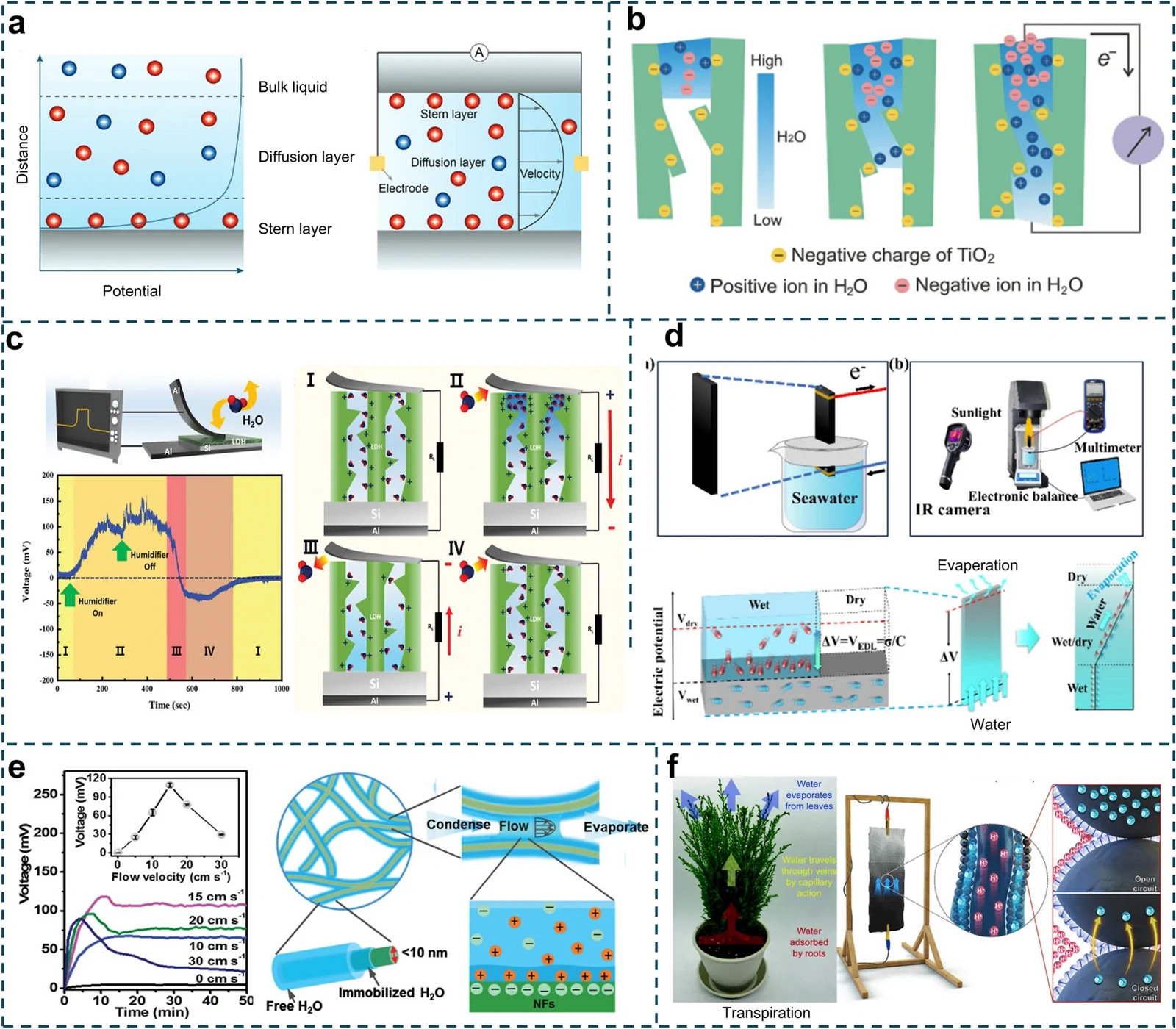
Moisture electricity generation based on streaming potential mechanism. **a** EDL of the solid-water interfaces. Reproduced with permission [9]. Copyright 2022, American Chemical Society. **b** Protons diffusion together with water molecules along the charged walls when moisture penetrate inside nanowire film. Reproduced with permission [65]. Copyright 2018, Wiley–VCH. **c** Convective electric current along the flow direction. Reproduced with permission [85]. Copyright 2018, Wiley–VCH. **d** Mechanism and model of hydroelectric power generation for water evaporation induced charge migration. Reproduced with permission [90]. Copyright 2024, Elsevier. **e** Fibers and cloth under the continuous water flow to generate electricity through the streaming current mechanism. Reproduced with permission [92]. Copyright 2019, Wiley–VCH. **f** Fabric with salt to increase the water absorption and ions flow through the channels for electricity generation. Reproduced with permission [93]. Copyright 2020, Royal Society of Chemistry

The streaming potential mechanism involves the conversion of fluid motion induced by water evaporation into electricity based on electrokinetic effects. When a polar liquid like water flows through narrow channels or porous materials with fixed surface charges, the fluid can drag counter-ions from the electric double layer along with it and generate an electric current. This is the classical streaming current phenomenon from electrokinetics (Fig. 3a) [9]. In a solid channel with charged walls, an EDL can be formed at the water–solid interface as the surface charge attracts a layer of oppositely charged ions in the fluid, forming a diffuse layer of ions extends into the channel which is also called by the Debye layer (Fig. 3b) [65]. If the channel diameter is comparable to or smaller than twice the Debye length, the EDLs from opposite walls overlap and the entire cross section of the channel has a region with net charges [79–84]. Thus, when water is driven to flow through such a nanoscale or microscale channel with the help of a pressure difference or by capillary action due to evaporation at one end, it carries the ions flow together. Thus, the movement of these ions result in a convective electric current along the flow direction and an equally opposite charge accumulates at the channel ends, leading to a measurable voltage difference between the inlet and outlet of the channel (Fig. 3c) [85]. This voltage is known as the streaming potential, and the corresponding current is the streaming current, which can be similarly described by the Helmholtz–Smoluchowski equation in fluidic systems. In more complex porous networks, one must consider the distribution of pore sizes and lengths [86–89]. Recent multiphysics simulations have incorporated evaporation dynamics as a boundary condition driving flow, enabling more accurate prediction of output under ambient conditions (Fig. 3d) [90]. For instance, Su et al. developed a comprehensive model for a hydroelectric nanogenerator that coupled Darcy flow, vapor diffusion, and Nernst–Planck ion transport [91]. Their simulation treated the evaporative flux at the outlet as a streaming potential boundary condition and successfully reproduced experimental trends, providing microscopic validation of how streaming currents arise from evaporation [91]. The models highlight the fact that in real material with functional groups, some fraction of the generated current can come from ions diffusing in addition to streaming convection, especially initially when the materials are first wetted. Indeed, many experiments have observed that both mechanisms can operate simultaneously.

Streaming potential enables continuous electricity generation as long as a steady moisture flux is maintained. In a typical streaming-based MEG, one end of a porous material is connected to a water source or a high-humidity environment, and water permeates into the porous network by capillary forces. At the other end, water exits either by evaporation into drier air or into an absorbent, creating a quasi-steady flow through the material (Fig. 3e) [92]. The flowing water interacting with charged pore surfaces produces a constant ionic flow. Importantly, streaming currents do not necessarily rely on any chemical dissociation of surface groups unlike the ion diffusion mechanism. On the contrary, they can occur even with an inert charged surface in an ionic solution. Thus, streaming-based generators tend to produce a stable DC output as long as the moisture gradient or water flow persists (Fig. 3f) [93]. For example, it has been shown that a continuous voltage of ~ 1 V can be obtained for hours or days without apparent drop by maintaining water evaporation from a reservoir [58]. As the output current in streaming based MEGs is related to the flow rate and the net charge density carried by the flow, higher humidity gradients and evaporation rates can increase the flow rate, as well as materials with higher surface charge can increase the output [32, 65]. One significant feature is that streaming currents typically require an open and percolating pathway for water flow. If the material dries out completely, the generation stops. Thus, streaming MEGs often incorporate a continuous water supply or an environment where one side is constantly contact liquid water or a hydrogel [66, 83, 94–96].

### Factors Affecting Output Performance

The output electricity performance is a key aspect for the practical applications of MEG in different scenarios. As the understanding of mechanism for electricity generations goes deeper, the design and tunability of the MEG for enhancing output has been improved. As the electricity generation process is quite complex, there are many factors influencing the eventual output, including the functional material properties, device structures and external environmental conditions.

The material property is the primary factor to control the electricity output. High ionic conductivity and abundant ionizable functional groups are crucial for high MEG output [5]. Functional materials with plentiful surface charges form strong electric double layers or releasing more mobile charges when in contact with water. Similarly, materials must be highly hydrophilic: polar or hygroscopic polymers (such as polyvinyl alcohol-alginate hydrogels) or salts (e.g., LiCl, CaCl_2_) absorb moisture and release mobile ions. For example, a PVA–alginate hydrogel MEG outperforms neutral PVA because the alginate’s dissociable Na^+^/Cl^−^ greatly increases ion availability [49]. Increased porosity and nanopore volume also boost performance by providing more pathways for water and ions. In one study a CNT/AAO device used 90 nm pores (comparable to the Debye length) to optimize ion rectification; pores too large or too small reduced output [97]. Thus, materials should be water-wetting and ion-conducting (high ionic conductivity, rich in charged functional groups, and nanoporous) to maximize moisture uptake and ion transport.

The device structure is another factor influencing the output capabilities. Architectures that enforce directional water/ion flow substantially increase output. Thin-layer or nanofluidic devices shorten diffusion paths, whereas thicker materials slow diffusion. Interface asymmetry is often exploited. Bilayer or gradient structures create internal moisture gradients that drive ion migration. For instance, a step-graded polysaccharide film (multilayer with varying ionic group density) exhibited 10 times higher voltage and current than a uniform film by promoting unidirectional ion diffusion [52]. A hygroscopic/evaporative bilayer (LiCl‐doped cellulose plus carbon-black cellulose) sustained a continuous water flow from one side to the other [52]. In ionic-diodes, mismatched surface charge (negative CNT vs. positive AAO) produces a built-in field that pumps ions [38]. Surface area is also key: 3D architectures or textured electrodes (e.g., high-surface-area fabrics, etched Si nanowires) provide more active sites and faster evaporation [98]. In one device an added absorbed layer both captured moisture and maintained conductivity, yielding a stable 0.72  V output over ~ 50 days [52]. Large-area or series/parallel stacking also scale current and voltage for practical loads [55]. Thus, device design, including layer thickness, porosity, surface-area, and electrode asymmetry, strongly modulates ion transport and hence MEG power.

Ambient humidity, temperature and airflow directly affect MEG performance. Higher relative humidity generally increases output by supplying more water for ion generation [13, 27, 65]. For example, one study found the open-circuit voltage and short-circuit current of a MEG rose monotonically as RH went from 10% to 93% [16]. Temperature has a dual effect: warming accelerates evaporation, sustaining moisture gradients and boosting output, whereas cooler conditions slow evaporation [9, 11, 26]. Indeed, experiments showed that raising temperature increases current density due to enhanced evaporation and capillary flow. Conversely, very high humidity can collapse gradients. At near 100% RH, devices may saturate and current eventually falls off [12]. Airflow (or wind) also enhances evaporation. Gentle air flow can refresh the evaporative surface and maintain steady water flux [26]. Finally, imposed humidity gradients are often necessary for continuous output [99]. Thus, environmental factors, including RH, temperature, and airflow critically determine the sustained voltage/current of MEGs, and must be optimized or engineered for practical applications.

## Material Innovations

Harvesting electricity from ambient humidity has developed rapidly due to the significant innovations of materials for MEG in recent years. Early MEGs relied on simple hygroscopic films such graphene oxide and produced only intermittent, low-grade outputs on the order of millivolts [20]. Since then, advances in material science have dramatically improved performance through new materials and structural designs [71, 80, 100–103]. Researchers have demonstrated continuous, self-sustained electricity generation using diverse materials that maintain persistent moisture gradients, from synthetic polymers and hydrogels with rich ionic functional groups to bio-derived substances like protein nanofibers [27]. Open-circuit voltages have accordingly increased by orders of magnitude from tens of millivolts to the order of 1–2 V for single device. The total voltage can even reach up to hundreds of volts when multiple MEG units are integrated in series [104]. However, achieving practical power levels remains challenging due to the low current density output. Until recently, most MEGs delivered currents only in microampere-scale (often < 10 µA cm^−2^).

A key design trade-off lies in maximizing water absorption while preserving a moisture gradient across the device. On the one hand, stronger moisture absorption yields more ions and higher chemical potential energy and corresponding higher electricity output. On the other hand, excessive water can equilibrate the system and collapse the gradient that drives ion diffusion quickly. Thus, the material innovations focus on balancing hygroscopicity and asymmetry: enhancing water capture through surface chemistry and porosity, while maintaining an internal gradient via gradient architectures or heterogeneous compositions. State-of-the-art MEGs often combine strategies to ensure sustained outputs. For example, composite films of hydrophilic and less-hydrophilic layers have been designed to promote directional water transport. Also, recent studies also emphasize biologically materials for constructing MEG devices to meet the requirement of sustainable and green life. In this section, we discuss the major material classes enabling these advances including carbon-based materials, polymeric (hydrogel) materials, bio-inspired materials, oxides, and composite hybrid material systems. A brief outline of MEGs with different material systems is summarized in Table 2.

**Table 2:** Summary of MEGs with different material systems

Material	Functional layer	Mechanism	Voltage (V)	Current (µA cm^−2^)	Duration	Condition	References
Carbon-based material	Graphene oxide	Ion diffusion	0.02	6	5 s	30% RH	[20]
Carbon-based material	Graphene oxide	Ion diffusion	1.5	0.027	< 100 s	80% RH	[25]
Carbon-based material	Graphene quantum dot	Ion diffusion	0.15	1500	5 s	70% RH	[105]
Carbon-based material	Graphene oxide	Ion diffusion	0.07	1200	10 s	60% RH	[106]
Carbon-based material	Fluorinated graphdiyne	Ion diffusion	0.65	65	15 h	90% RH	[107]
Carbon-based material	Carbon black	Streaming current	1	< 0.1	160 h	DI water	[26]
Polymer	PSSA	Ion diffusion	0.8	100	1400 s	80% RH	[47]
Polymer	PSSA/PDDA	Ion diffusion	1.38	0.076	250 h	85% RH	[15]
Polymer	PVA/AlgCa/Na	Ion diffusion	1.3	1310	120 h	70% RH	[49]
Polymer	PAM	Streaming current	0.6	1160	360 h	90% RH	[57]
Polymer	PGA/CA	Ion diffusion	0.55	130	72 h	90% RH	[12]
Bio-derived material	Geobacter sulfurreducens	Streaming current	0.5	0.17	1500 s	50% RH	[27]
Bio-derived material	whey protein	Ion diffusion	1.45	113	–	40% RH	[37]
Bio-derived material	DNA	Ion diffusion	0.3	1.2	20 h	90% RH	[50]
Bio-derived material	Wood	Ion diffusion	0.57	4	25 h	85% RH	[104]
Bio-derived material	Lotus leaf	Streaming current	0.25	–	35 h	DI water	[39]
Oxide-based material	TiO_2_	Streaming current	0.5	8	100 s	80% RH	[65]
Oxide-based material	Al_2_O_3_	Streaming current	0.07	6.24	–	Salt water	[108]
Oxide-based material	ZnO/GO	Ion diffusion	0.4	50	8 h	90% RH	[109]
Composite materials	polymer–hydrogel–carbon	Streaming current	1.86	21.8	–	82.9% RH	[110]
Composite materials	Hydrogel/graphene oxide	Ion diffusion	0.6	1	120 h	80% RH	[76]
Composite materials	LiCl/carbon dopped cellules	Streaming current	0.78	0.8	240 h	50% RH	[55]

### Carbon-Based Materials

**Fig. 4:**
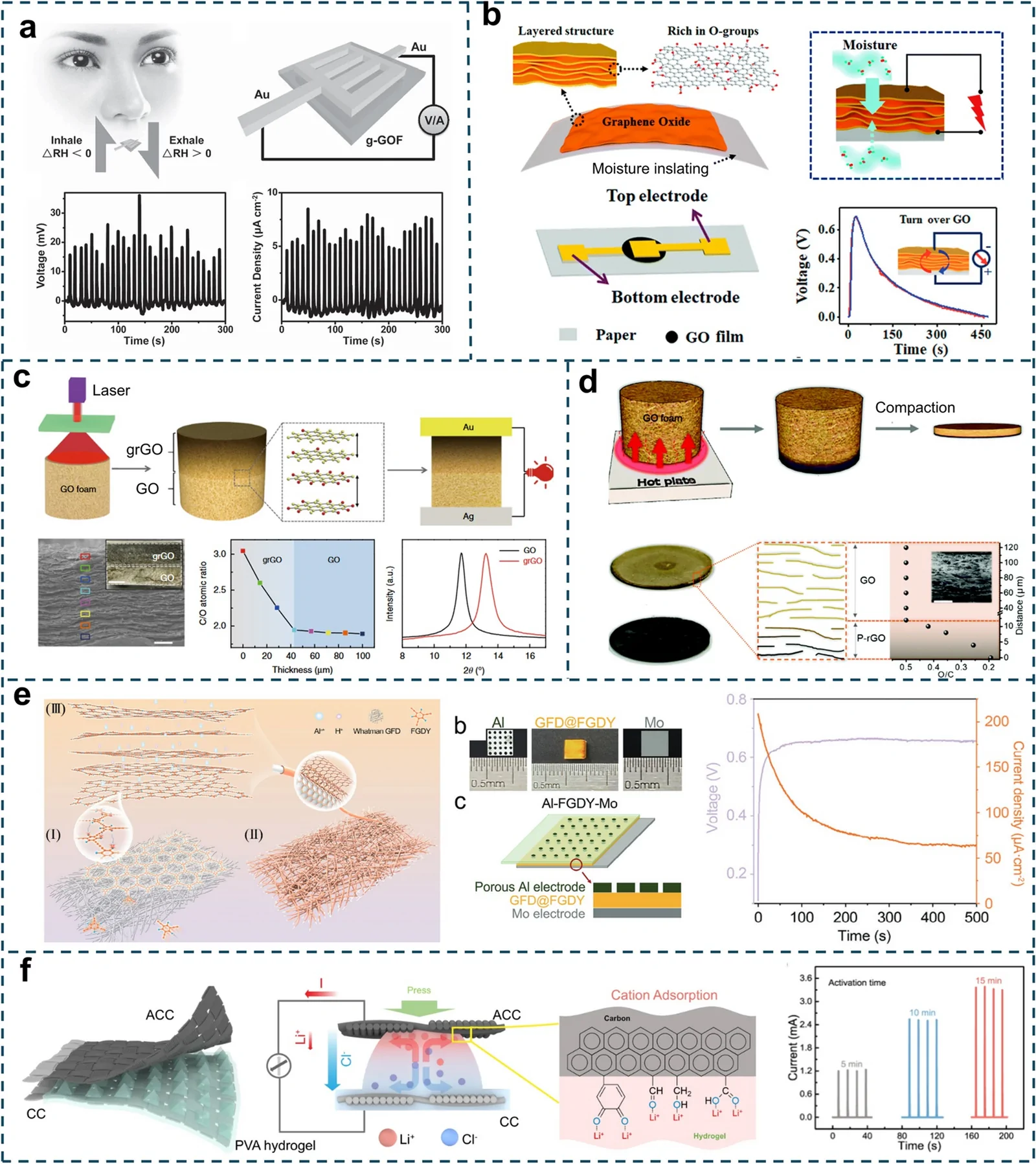
Carbon-based materials for MEGs. **a** Harvesting moisture from human breath based on GO film. Reproduced with permission [20]. Copyright 2015, Wiley–VCH. **b** Asymmetrical moisturizing of printed GO film. Reproduced with permission [99]. Copyright 2018, Royal Society of Chemistry. **c** Optimizing the GO/electrode interface by laser reduction of GO with vertical gradient. Reproduced with permission [25]. Copyright 2018, Springer Nature. **d** Internal gradient creation of a GO cylinder by chemical reduction. Reproduced with permission [33]. Copyright 2018, Royal Society of Chemistry. **e** Fluorinated graphdiyne film coordinated with aluminum ions as charge carriers for MEG. Reproduced with permission [107]. Copyright 2025, Wiley–VCH. **f** Porous carbon papers as electrodes to collect charge from a moisture-active layer. Reproduced with permission [115]. Copyright 2024, Springer Nature

Carbon-based materials have been at the focus of MEG research since the earliest demonstrations. GO films have been widely used as foundational material for MEGs design due to their abundant oxygen-containing functional groups (such as -COOH) and high water affinity [25, 41, 105, 106, 111]. In 2015, the direct power generation from a GO film under a moisture gradient was reported (Fig. 4a) [20]. GO can absorb moisture from air and dissociate it into mobile ions (mainly protons) under the interaction with hydrophilic species, establishing an ionic gradient along the film which produces the electricity output. This lays the foundation of the gradient-structure MEG concept, with one side of a hygroscopic carbon film exposed to moisture while the opposite side remains relatively drier, creating a sustained chemical potential difference. Subsequent graphene-based MEGs improved on this design. For example, Liang et al. achieved ~ 0.7 V output by asymmetrically moisturizing a printed GO film (Fig. 4b) [99], and Huang et al. engineered an interface-mediated GO device that reached ~ 1.5 V output, a remarkable jump enabled by optimizing the GO/electrode interface and film thickness (Fig. 4c) [25]. These studies underlined the role of surface modification that by tuning GO oxygen content and functional group gradient, the ionic diffusion and potential across the film can be improved.

Reduced graphene oxide (rGO) and partially functionalized graphene have been explored to introduce charge gradients. It was demonstrated that a thermally gradient-reduced GO where one end of a GO cylinder was chemically reduced to create an internal gradient of oxygen groups (Fig. 4d) [33]. This spontaneously produced an open-circuit voltage of ~ 0.6 V in ambient air without any external moisture gradient, as the built-in material asymmetry induced ion gradient and migration. Similarly, graphene nanostructures with spatially inhomogeneous functionalization by laser irradiation have been shown to sustain voltages of order 0.1–0.5 V by internal ion separation [112, 113]. Another two-dimensional carbon, graphdiyne (GDY), has recently emerged as a high-performance MEG material. Graphdiyne is a π-conjugated carbon network with inherent nanopores, which can be chemically modified to enhance ion transport. This GDY-based moisture cell achieved an exceptionally high mass-specific power density of ~ 371 µW g^−1^, with a stable ~ 0.65 V output sustained for 15 h (Fig. 4e) [107]. The impressive performance was attributed to large pore channels that reduced ion diffusion barriers, together with the hard acid–base interaction between Al^3+^ ions and fluorine on GDY that increased ionic conductivity. This example showcases how molecular-level engineering of carbon frameworks through dopants and functional groups can improve the electricity output.

Carbon nanotubes (CNTs) are another carbon-based material for electricity generation with water flow or evaporation through CNT networks for charge separation [114]. While CNTs alone are less commonly used as the pure active layer in recent MEGs, they frequently combine with other materials to improve conductivity and nanochannels. For instance, a study employed a hierarchically porous carbon nanomaterial with CNTs and nano-Al_2_O_3_ in a fibrous membrane to create a nanofluidic ionic diode generator [60]. In this device, modulated CNT networks lined the nanopores, yielding an open-circuit voltage of ~ 1.03 V from a single MEG device. The CNTs provided pathways for directional water transport, while the oxide nanoparticles enhanced the asymmetry of ion diffusion. Thus, the carbon nanomaterials serve to increase the surface area and charge-storage capacity of MEG films. In addition to CNTs, porous carbon papers or fabrics have been used as electrodes to collect charge from a moisture-active layer due to the good conductivity and flexibility (Fig. 4f) [115]. The high electronic conductivity of carbon ensures that electrons liberated by ion diffusion can efficiently flow through the external circuit, minimizing internal resistance losses. Carbon materials are also lightweight, flexible, and chemically tunable, making them attractive for wearable or printable MEG applications.

In terms of electrical performance, the carbon-based MEGs now have been developed rapidly. Graphene MEGs initially produced only tens of millivolts, but interface engineering and multilayer stacking can increase the voltage to ~ 1 V. The combination of GO with other functional materials has enabled high voltages. For example, a printed GO-based cell with silver electrodes inspired by the electric eel’s organ achieved 1.2 V. By printing 175 such cells in series, a stack voltage of ~ 192 V was obtained [116]. However, carbon MEGs often have moderate current densities around nA cm^−1^ to several µA cm^−1^ unless augmented by ionic polymers or electrolytes. Long-term stability can be also an issue. Researchers tried to address this by crosslinking GO or combining it with more stable matrices. Overall, carbon-based materials offer a versatile platform for MEGs, with tunable chemistry and form factors that have yielded many record performances. The continued development of novel carbon structures and their hybridization with other components is expected to further enhance the electrical output and durability of MEGs.

### Polymeric Materials and Hydrogels

Polymeric materials, especially hydrophilic hydrogels and polyelectrolyte membranes, represent another key class of moisture-electric generators. These materials contain abundant ionic functional groups such as -COO^−^, -SO_3_^−^, and -NR_3_^+^, which enable high water absorption and provide abundant charge carriers for electricity generation [56, 117–122]. It was demonstrated that a thin polymeric membrane with functional groups of -COOH could continuously generate power under ambient humidity by ion diffusion mechanism [14, 18, 18, 42, 114, 123–125]. When one side of such a membrane is exposed to moisture, water uptake causes mobile ions to redistribute across the thickness, establishing an electric potential. The use of ion-exchange membranes was shown to yield voltages of a few hundred millivolts when the ion diffusion was driven by unbalanced moisture in a humid environment.

**Fig. 5:**
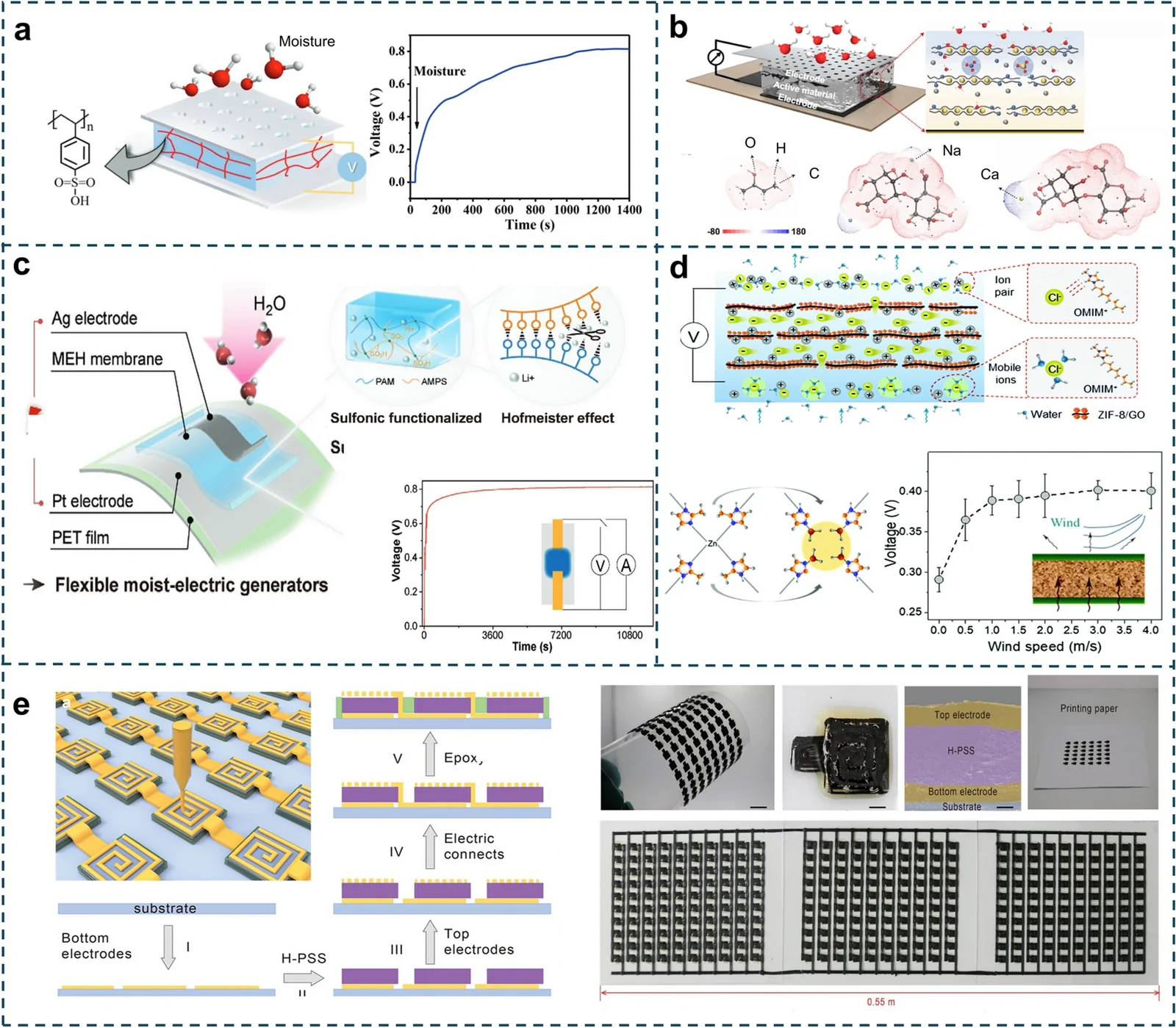
Polymeric materials and hydrogels for MEGs. **a** Polymer-based MEG comprising a sulfonated polyelectrolyte layer could generate ~ 0.8 V. Reproduced with permission [47]. Copyright 2019, Royal Society of Chemistry. **b** Hydrogel based MEG can produce milliamp level current under moisture. Reproduced with permission [49]. Copyright 2024, Springer Nature. **c** Modification of dynamics of ions and charges migration by ions doping in hydrogel. Reproduced with permission [34]. Copyright 2023, Wiley–VCH. **d** Polymer–nanochannel composite to enhance selective ion transport. Reproduced with permission [128]. Copyright 2022, Royal Society of Chemistry. **e** 3D printed MEG based on the acid-doped polyelectrolyte membrane. Reproduced with permission [130]. Copyright 2023, Wiley–VCH

A breakthrough in this category came with the development of hydrogel for constructing MEGs. Hydrogels are polymer networks with a huge water absorption capability, and by doping them with salts or ionic monomers, they can serve as efficient electricity generators. It was reported that a polymer-based MEG comprising a sulfonated polyelectrolyte layer that could generate ~ 0.8 V in ambient air (Fig. 5a) [47]. The device was essentially built with an ionic hydrogel film where moisture adsorption induced a continuous diffusion of ions toward one electrode. Building on this, a bilayer polyelectrolyte film system was built to achieve high voltages under moist environment. A cationic polymer layer was stacked with an anionic polymer layer to create an internal ionic junction. In ambient air (~ 25% RH), a stack of these bilayer films produced up to ~ 1000 V open-circuit output by connecting hundreds of MEG devices in series [15].

Contemporary research on polymeric MEGs has focused on hydrogels with tailored composition to boost ionic conductivity and water absorption. By constructing a supramolecular network of PVA and sodium alginate, a natural anionic polymer, crosslinked with polyvalent ions, a hydrogel with extremely high moisture absorption capacity and a slow-release water gradient was obtained. The PVA/alginate matrix holds a large amount of moisture and retains a gradient of water content across its thickness. Additionally, the presence of abundant dissociable ions (Na^+^ from alginate) and the continuous ionic pathways facilitate a strong diffusion current. As result, a single piece of this hydrogel generates open-circuit of ~ 1.3 V, with a short-circuit current density of ~ 1310 µA cm^−2^. This milliamp-scale output far surpasses most MEGs reported. Arrays of the hydrogel in parallel delivered about 65 mA current, which is enough to power a digital clock, light up LEDs, or even charge a smartwatch in ambient conditions (Fig. 5b) [49].

Doping the polymers or hydrogels with ions by adding hygroscopic salts such as LiCl or CaCl_2_ is the one effective method to modify the dynamics of ions and charges migration. The salts act as a water sorbent and in some cases also provide mobile ions (Fig. 5c) [34, 126]. Polyamines and polyelectrolyte complexes have also been used to create asymmetric ion distributions. A capacitor-inspired MEG was reported where a breathable polyamide nanofiber membrane was coated with charged polymers, yielding a durable device with improved stability over 10,000 humidity cycles [127]. A significant innovation in polymeric MEGs is the use of thermoresponsive and stimuli-responsive hydrogels to regulate moisture transport. For example, a hydrogel of poly(N-isopropylacrylamide) (PNIPAM) was used to cyclically absorb and release water with temperature changes, effectively acting as an autonomous moisture pump to sustain a gradient, with an output voltage of 430 mV lasting for more than 15 days. While PNIPAM alone produces this continuous output, coupling such dynamic polymers with MEG circuits could smooth out fluctuations in humidity [57]. Another approach of polymeric modification is designing defect-rich ionic composites. Lv et al. introduced defects into a polymer–nanochannel composite to enhance selective ion transport, boosting the efficiency of moisture-to-electric conversion (Fig. 5d) [128]. By creating specific ion pathways and blocking counter-charge recombination, output current and long-term stability of the device can be improved. As a result, an output current of 1,500 µA cm^−2^ for more than 80 h when it is placed under a humidity gradient of 56% can be obtained, contributing to a power density of 109.2 μW cm^−2^.

From a point of fabrication, polymer MEGs have advantages of simplicity and scalability over other materials. Polymers are usually made by solution casting, printing, or freeze-drying, the procedure of which can be easily assembled into large-area production. For example, a freeze-dried bilayer of sodium alginate and carbon nanotubes was made in one step to yield an asymmetric film [129]. Likewise, inkjet printing or 3D printing can pattern polyelectrolyte inks to form multiple MEG cells in series on flexible substrates (Fig. 5e) [130]. This highlights how polymer-based materials can be integrated in advanced manufacturing for practical devices. They combine high moisture affinity, mechanical flexibility, and easy processability. Challenges remain in preventing performance degradation over long durations and in further scaling up the power output.

### Bio-Derived Materials

**Fig. 6:**
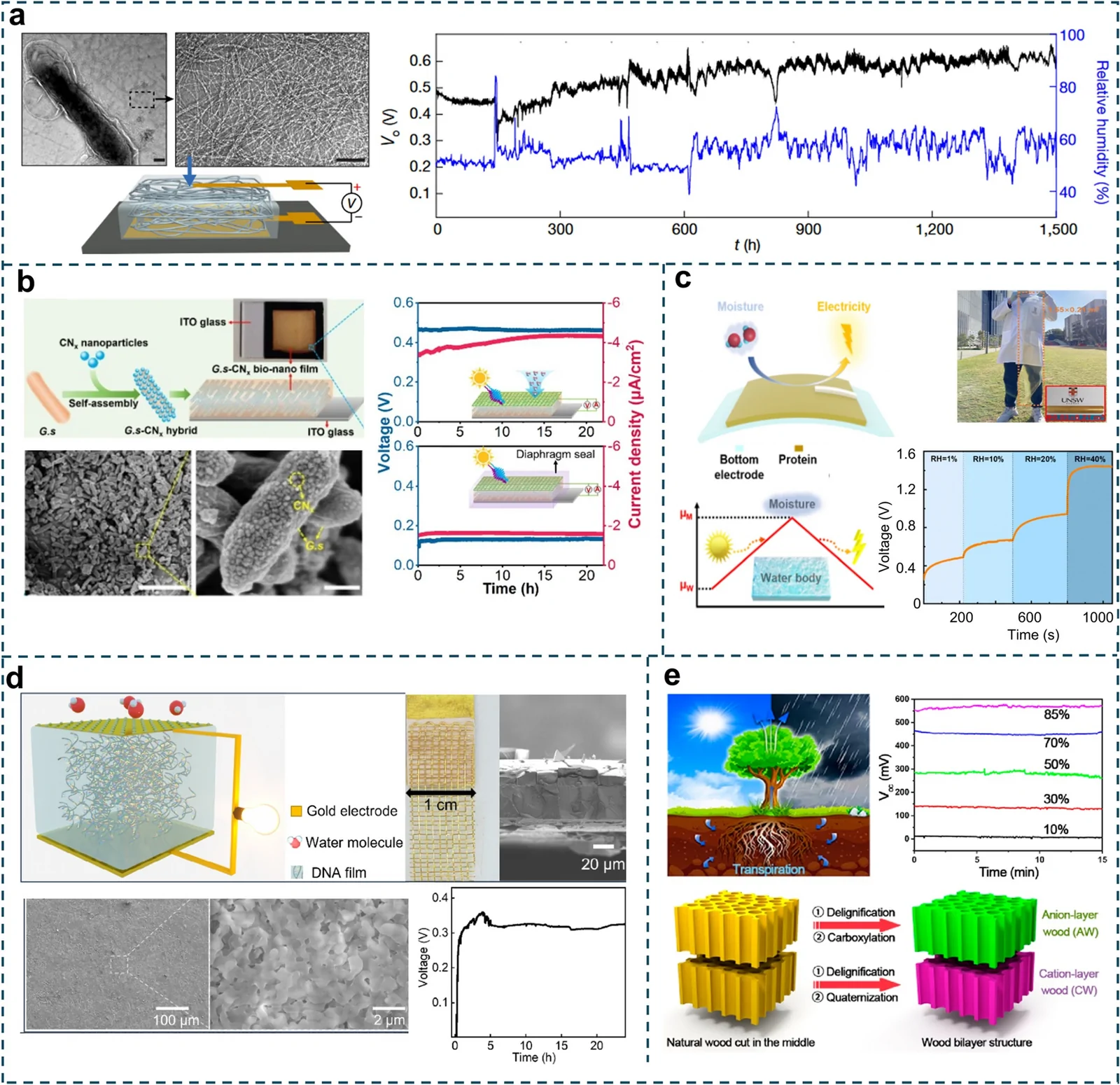
Bio-derived materials for MEGs. **a** A thin film of microbial protein nanowires can continuously produce electricity from humidity in air over 1500 h. Reproduced with permission n[27]. Copyright 2020, Springer Nature. **b** Porous network based on bacterium Geobacter sulfurreducens for MEG. Reproduced with permission [131]. Copyright 2023, Elsevier. **c** Cheap and abundant protein mixture from dairy for MEG. Reproduced with permission [37]. Copyright 2023, Royal Society of Chemistry. **d** DNA-based recyclable moist-electric generator. Reproduced with permission [50]. Copyright 2025, Tsinghua University Press. **e** Chemically treated natural wood for MEG. Reproduced with permission [104]. Copyright 2022, American Chemical Society

Bio-derived materials have introduced a fascinating option for moisture electric generation by taking clues from natural hygroscopic systems and leveraging biomolecules as functional components. They have expanded the MEG field by introducing naturally abundant and eco-friendly materials capable of converting moisture energy into electricity. A key achievement in this area was the discovery that a thin film of microbial protein nanowires can continuously produce electricity from humidity in air over 1,500 h (Fig. 6a) [27]. It was reported that under exposed to ambient moisture, nanowires based on bacterium Geobacter sulfurreducens form a porous network can generate a voltage continuously (Fig. 6b) [131]. In an Air-Gen device, a ~ 7 µm thick film of Geobacter nanowires produced a continuous 0.4–0.5 V and ~ 17 µA cm^−2^ current in 45% RH air [132]. It was considered that the top of the film adsorbs water and releases protons that migrate downward through the protein network, creating an electrochemical potential. Because the nanowire film naturally had a gradient in pore size, it maintained a moisture differential between two sides as long as humidity was present. This work demonstrated for the first time that a spontaneously sustained moist electricity can be realized using a material derived from biology.

Inspired by the protein nanowire device, other more accessible bio-materials was also used for constructing MEGs. One successful candidate is whey protein, a cheap and abundant protein mixture from dairy can achieve surprisingly high outputs (Fig. 6c) [37]. By casting and drying whey protein into thin films and chemically tuning surface charge, an open-circuit voltage up to 1.45 V at 40% RH can be obtained. This is one of the highest voltages for single MEG device. The whey protein MEG was also extremely low-cost and showed self-healing behavior under moisture, with small cracks in the film would re-bind due to the supramolecular interactions in the protein. Furthermore, the MEG device with protein film can operate across harsh conditions such as a desert environment with RH as low as 26% [37]. Another intriguing biomaterial for MEGs is DNA. Guang et al. demonstrated a recyclable MEG using salmon DNA to form a porous membrane (Fig. 6d) [50]. In the device configuration, DNA is a polyanion with phosphate backbone and can coordinate cations. When assembled into a membrane through freeze-drying, it creates a sponge-like film capable of attracting moisture. The DNA-MEG produced a stable voltage of ~ 0.3 V with a current density around 1.2 µA cm^−2^ at 90% RH [50]. While the output is not high, an interesting property is full recyclability as the DNA film can be completely dissolved in water and re-cast into a new film without performance loss, an unique property rarely seen in inorganic or synthetic materials.

Silk-protein, possessing a unique hierarchical structure consisting linear polypeptide chains formed by amino acids linked by peptide bonds [133, 134], is also an interesting natural for building MEG devices. A MEG with pure silk-protein is able to produce an output voltage of 0.15 V in the ambient moist environment [132]. By increasing the film thickness to improve the water absorption gradient, it was shown that the output voltage can be increased from 0.1 to 0.26 V. To further enhance the output, the electrospun film with oxidized silk-protein and Ag nanoparticles can increase the positive ions [135]. Thus the output voltage and current can be increased to 0.28 V and 0.19 µA cm^−2^, respectively. In the meantime, by adding sericin that contains over 70% polarized side-chain amino acids into the silk-protein, the hygroscopic property was largely enhanced, which is promising for enhancing the output [36]. Furthermore, the transport behaviors can be modified to produce the asymmetric ions flow through the bilayers design [136]. When exposed to moisture, the hydratedated and positively charged nanochannels can create ion gradient, with optimal voltage of 0.121 V. The silk-protein can be chemically modified with negatively charged sulfonic acid groups to enhance the ions selectivity for electricity output [137]. As a result, a maximum voltage of 0.441 V can be obtained, demonstrating the great potential for energy harvesting using silk-protein material.

In addition to the bio-derived materials designed, natural hygroscopic structures are also used for directly building MEG devices. For instance, the hierarchical design of wood and plant tissues that transport water has been used for MEG designs. A notable example is ionically conductive wood by carbonizing or chemically treating natural wood to create a moisture path that mimics tree transpiration to generate electricity (Fig. 6e) [104]. These wood-based MEGs benefit from the intrinsic channels of wood that promote directional moisture flow and can yield voltages on the order of tens of millivolts per cm of length. Although the output is not as high as engineered polymers, such systems are promising due to their good sustainability and easy accessibility. In addition to the natural wood, lotus leaf was also used for constructing MEG devices, enabling direct harvest of latent energy via leaf transpiration [39]. The sustained all-day electricity generation was demonstrated. An open-circuit voltage of 0.25 V and a short-circuit current of 50 nA can be obtained, which was attributed to enhanced transpiration rate, stomatal conductivity and temperature. This study provides a fresh perspective for advancing green energy technologies through the lotus leaf transportation in nature.

### Oxide-Based Materials

**Fig. 7:**
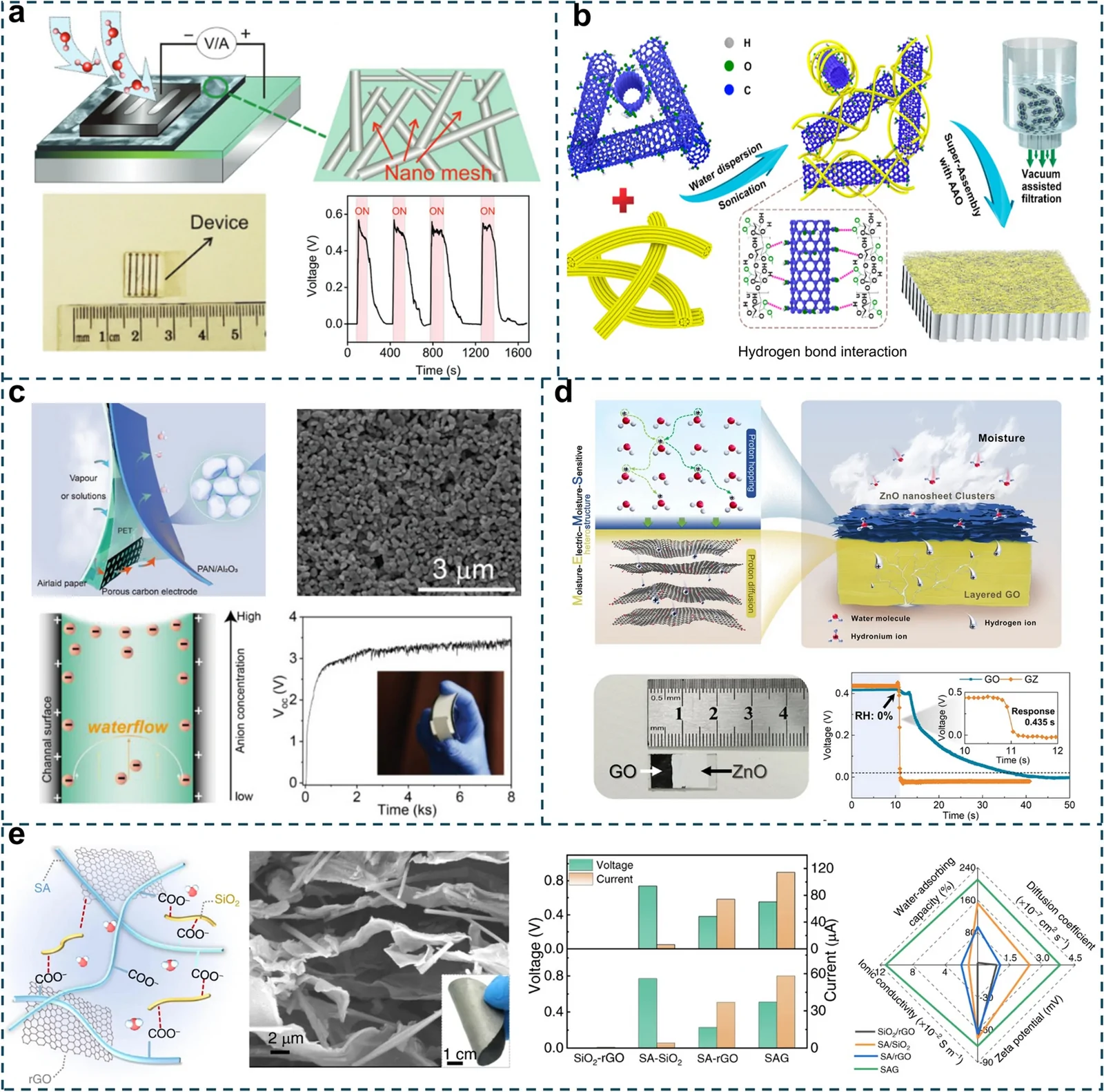
Oxide-Based Materials for MEGs. **a** Sandwiched TiO_2_ nanowire networks with nanopores for water diffusion and electricity generation. Reproduced with permission [65]. Copyright 2018, Wiley–VCH. **b** Fiber-based nanofluidic diode by coating an AAO membrane on a textile fiber allows water vapor to pass and condense. Reproduced with permission [108]. Copyright 2023, American Chemical Society. **c** A porous film with Al_2_O_3_ nanoparticles for electricity generation. Reproduced with permission [80]. Copyright 2023, Wiley–VCH. **d** Graphene oxide combined with a layer of ZnO nanowires for electricity generation. Reproduced with permission [109]. Copyright 2024, Springer Nature. **e** Surface modification using sodium alginate, silicon dioxide nanofiber, and reduced graphene oxide for improving output. Reproduced with permission [40]. Copyright 2022, Springer Nature

Oxide-based materials, including metal oxides and other inorganic compounds, represent another category of MEG materials due to their good stability and ability to form nanostructures. Metal oxides typically have hydrophilic surfaces that can absorb water effectively, and some can exchange ions with the adsorbed moisture, creating a potential difference. For example, the sandwiched TiO_2_ nanowire networks with nanopores for water diffusion can produce voltage of 0.5 V with the interaction with moisture (Fig. 7a) [65]. As the nanowire networks have good mechanical properties, the TiO_2_ nanowire-based MEG has good flexibility, which can be used for constructing self-powered wearable electronics for health monitoring. Although many oxide-based materials have been used for harvesting energy from ambient moisture, most of these devices are based on streaming current mechanism, and the voltage can be as high as several volts. However, the current is usually low (typically in nA level) compared with the device with other materials.

One prominent approach involves nano-porous oxide membranes using anodic aluminum oxide (AAO), which has an array of aligned nanopores for moisture migration and inducing electric power. A fiber-based nanofluidic diode by coating an AAO membrane on a textile fiber allows water vapor to pass and condense (Fig. 7b) [108]. Due to surface charges on the pore walls, an asymmetric ion flow like a diode is established under a moisture gradient. This device was capable of multimodal energy harvesting with responding to moisture flow, liquid water, and even saline gradients. The inherent selective transport in charged nanopores leads to a streaming current as water percolates, generating voltages in the range of a few hundred millivolts. The use of polymer polyacrylonitrile (PAN) for strong tandem and binding of alumina Al_2_O_3_ nanoparticles can result in the formation of porous films with excellent flexibility and mechanical impact resistance, which can withstand bending of more than 180° and high-speed water flow impact of 9.92 m s^−1^ (Fig. 7c) [80]. As a result, the water-volt ion sensor based on the flexible and resilient PAN/Al_2_O_3_ film exhibits a maximum open-circuit voltage (in deionized water) of up to a record of 3.18 V. Further, through the structural design of the contact and non-contact moisture collection device, it was successfully applied to the construction of wearable multi-function sensor energy supply and self-actuated sweat electrolyte sensor, and realized the sports health monitoring based on the water-volt effect.

Zinc oxide (ZnO) has also been investigated, particularly in hybrid structures. ZnO is a wide-bandgap semiconductor that is piezoelectric and moisture-sensitive. A recent study constructed a GO-ZnO heterostructure MEG, where a layer of graphene oxide was combined with a layer of ZnO nanowires (Fig. 7d) [109]. The GO absorbed moisture and acted as the ion source, while the ZnO responded to the moisture by changing surface charge distribution. This moisture-sensitive heterojunction produced enhanced outputs when ambient humidity was increased. The GO provided a voltage from ion diffusion and the ZnO layer acting as a moisture-sensing resistor can modulate the current flow. Another interesting inorganic oxide for constructing MEGs is silicon nanostructures. When formed into nanowires or nanofilms, silicon can spontaneously generate electricity with moisture. Prior work has shown that a silicon wafer with nano-needle arrays could drive an ionic current by capillary condensation of water between the needles, yielding an output voltage. A properly surface-treated Si nanowire array can produce a continuous output. Although not as widely used as carbon or polymer, silicon-based MEGs could be integrated with conventional semiconductor technology, potentially enabling on-chip moisture-powered microdevices. Overall, oxide-based materials in MEGs contribute through structural features of porosity and channels, together with surface modification such as ion exchange and adsorption (Fig. 7e) [40]. They tend to be highly stable as many oxides are inert and can endure environmental extremes, which is advantageous for durability compared to most of other materials.

### Composite and Hybrid Materials

**Fig. 8:**
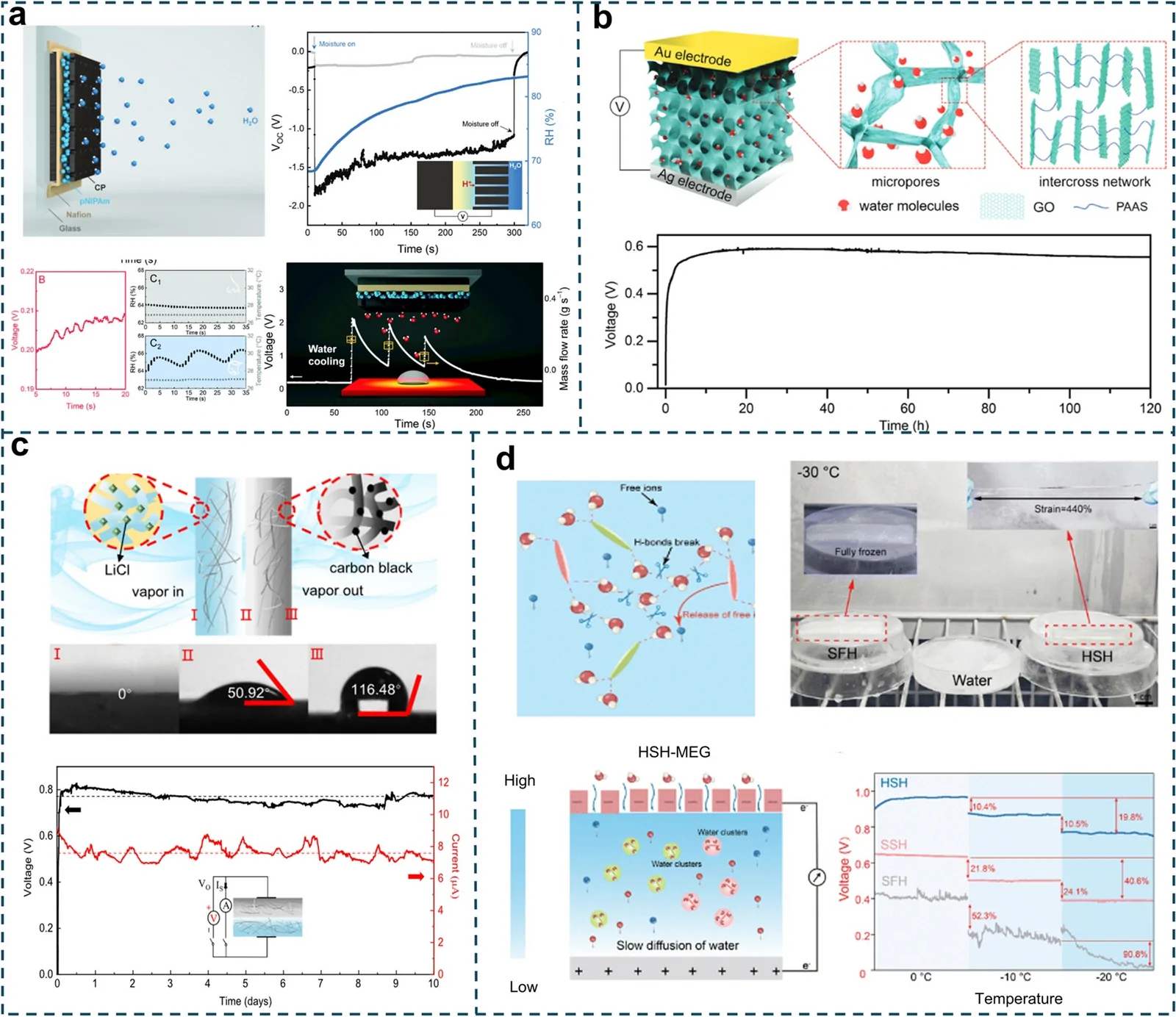
Composite and hybrid materials for MEGs. **a** Ionic polymer-hydrogel-carbon composite as a moisture energy harvester. Reproduced with permission [110]. Copyright 2022, Royal Society of Chemistry. **b** Graphene oxide composite film that could serve as an all-region applicable power source. Reproduced with permission [76]. Copyright 2019, Royal Society of Chemistry. **c** Self-sustained generator can be obtained by integrating two heterogeneous layers. Reproduced with permission [55]. Copyright 2022, Springer Nature. **d** Combination of components allows optimizing each aspect of the energy conversion chain. Reproduced with permission [138]. Copyright 2025, Royal Society of Chemistry

Although there are various hygroscopic functional materials systems that can be used for constructing MEG device, the single type of material usually lacks the enriched tunability of functional groups and ions migration. Composite materials take advantages of the strengths from multiple components, thus the materials and charges tunability can be largely improved through some synergistic effects between materials. As a result, the electricity performance can largely benefit from this hybridization. For MEGs, this often means combining an ionic conductor to generate charge from moisture with an electronic conductor to efficiently carry electrons, and sometimes a structural scaffold. One common example is the polymer–carbon composite, where polymers or hydrogels provide the ionic functionality while carbon nanomaterials provide conductivity. Liu et al. presented an ionic polymer–hydrogel–carbon composite as a moisture energy harvester (Fig. 8a) [110]. In their device, a polyacrylic-acid-based hydrogel was mixed with carbon particles, yielding a composite film with improved electron transport and moderate ion exchange capacity. When exposed to a moisture flow, this hybrid produced a voltage of ~ 0.5 V and higher currents than the pure hydrogel due to the low internal resistance from carbon network. Another example is graphene–polymer laminates. A graphene oxide composite film could serve as an all-region applicable power source (Fig. 8b) [76]. By integrating GO with a hygroscopic polymer, continuous output can be achieved even in fluctuating RH conditions as the polymer buffered the water. This composite delivered ~ 0.6 V and continuous current for over 120 h, demonstrating good environmental adaptability.

Layered composites are particularly effective at managing moisture gradients by taking advantages of the synergistic effects between layered materials. For example, a self-sustained generator can be obtained by integrating two heterogeneous layers, with one strongly absorbed moisture and one facilitated evaporation (Fig. 8c) [55]. A highly hygroscopic LiCl doped polymer was combined with more porous but less hygroscopic layer, ensuring that one side always remained wetter than the other to create a self-maintained cycle of adsorption and desorption. In such design, the structural integration of two layers results in the synergistic effects between layered materials to manipulate the moisture dynamics as well as the directional migration of charges continuously, which largely enhances the device performance. This device continuously produced power without needing an external dry environment. Such composites of different wettability materials can greatly improve stability and continuity. Hybridization can also be used for tuning ion transport for MEGs. For example, defect-engineered composites used a polymer matrix with ceramic nanoparticles that introduced selective ion channels [128]. By tuning the interface at these polymer–particle boundaries, preferential pathways that boosted the ionic diffusion while impeding backflow of counter-ions can be obtained [128]. In some cases, the ion gradient can also be directly formed within the composited layers. For example, by stacking two layers each functional materials capable of releasing positively and negatively charged free ions, respectively, the ions gradient and overall potential can be largely increased due to the dual ions migration [15].

Composite materials have achieved high Figs of merit in MEGs. The rational combination of components allows optimizing each aspect of the energy conversion chain (Fig. 8d) [138]. For example, the PVA–alginate hydrogel for moisture absorption and ion conduction, combined with a high-surface-area conductive carbon cloth yielded both high voltage and high current simultaneously [115]. In another case, electrospun nanofiber with abundant pores were coated with polyelectrolytes and carbon nanotubes to create a breathable, wearable MEG fabric [139]. This composite fabric could generate 0.1–0.2 V just from the humidity difference between skin and air, which was light and breathable enough for continuous wear. The composite approach played an important role as the nanofiber provided mechanical strength and breathability, while the polyelectrolyte provided ionic functionality and the CNTs imparted conductivity.

## Device Architectures

### Planar Film Devices

**Fig. 9:**
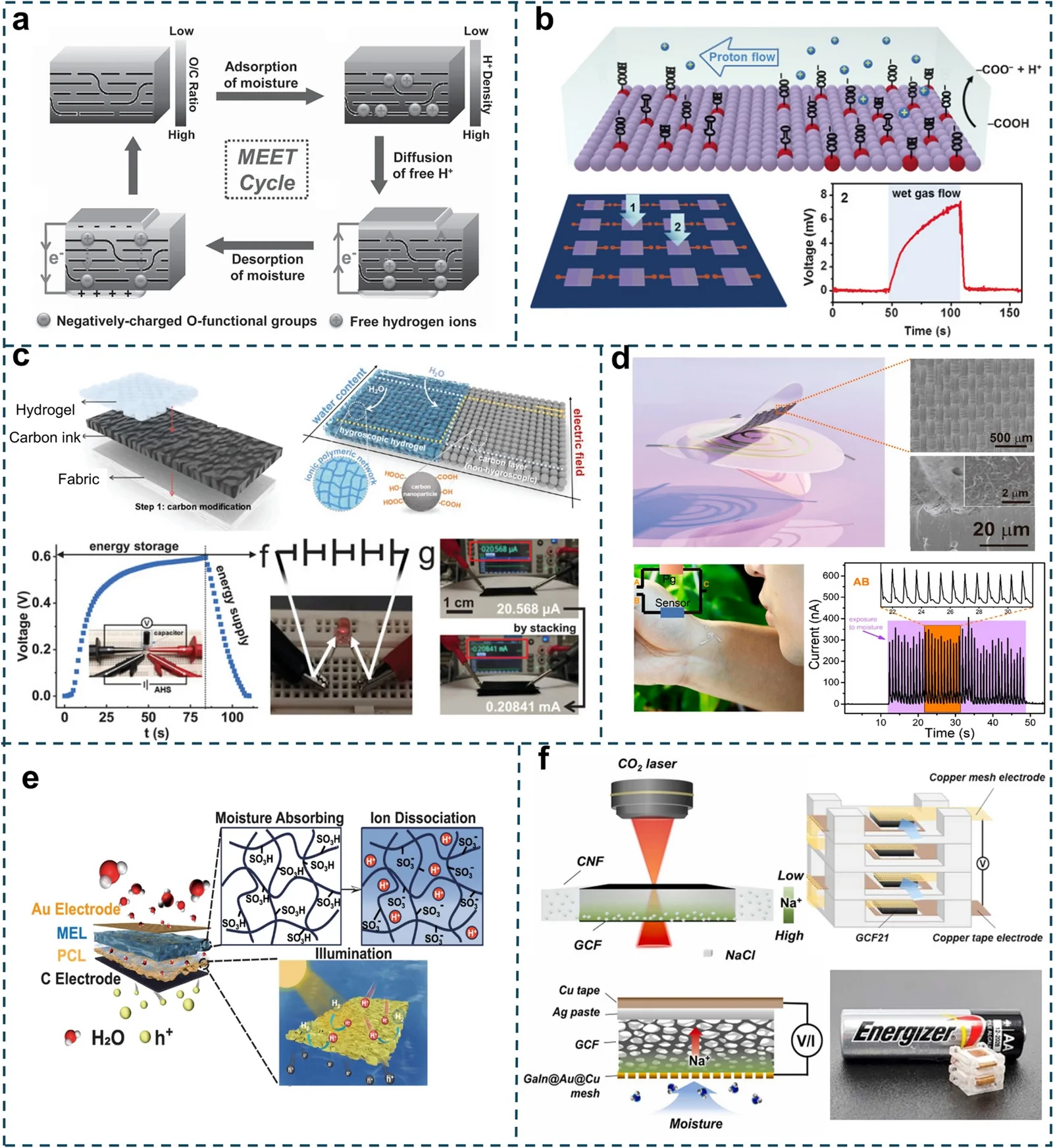
Planar Film Devices. **a** Single-layer graphene oxide film for planar MEG. Reproduced with permission [20]. Copyright 2015, Wiley–VCH. **b** Planar diffusion of protons in planar MEG. Reproduced with permission [140]. Copyright 2016, Wiley–VCH. **c** Hydrogel materials for planar MEG. Reproduced with permission [35]. Copyright 2022, Wiley–VCH. **d** Wearable planar MEG for health monitoring. Reproduced with permission [45]. Copyright 2019, American Chemical Society. **e** Ultra-thin film rapidly absorb moisture for electricity generation. Reproduced with permission [29]. Copyright 2025, Springer Nature. **f** MEG are scaled up to increase the total output. Reproduced with permission [112]. Copyright 2022, Elsevier

Planar moisture-electric generators typically consist of a thin film or membrane that harnesses a moisture gradient between two regions of surface, either from planar or vertical direction, to produce electricity. Planar film is also the widest structure used for MEG. In early examples, a single-layer graphene oxide film with an asymmetric distribution of oxygen functional groups was shown to generate about 0.035 V under ambient humidity (Fig. 9a) [20]. When exposed to moisture, the more hydrophilic region releases a higher concentration of protons, driving ion diffusion toward the drier side and produce voltage output (Fig. 9b) [140]. Subsequent studies dramatically improved the output of planar films into 0.4–0.7 V with 2–25 μA cm^−2^ of current density by optimizing materials surfaces and film thickness [141]. Similarly, a 7 μm-thick film of protein nanowires harvested from Geobacter microbes was shown to continuously generate 0.5 V in ambient air by adsorbing atmospheric humidity, which denoted as “Air-gen” device that remarkably works even in environments of extremely low relative humidity [27].

The materials for planar MEG films range widely, including carbon-based films (graphene oxide, carbon black, carbon nanotube networks), polymers, biomaterials, and oxides, have been extensively explored due to their high surface area and functional group tunability (Fig. 9c) [35]. Planar devices have demonstrated power densities on the order of 0.1–0.5 µW cm^−2^ in early designs [141], now improved to the μW–mW cm^−2^ range with material and structural optimizations (Fig. 9d) [45]. Typically, higher humidity and temperature can increase the output of MEG, however, some designs retain some output even at < 20% RH by using ultra-thin films that rapidly re-adsorb any available moisture (Fig. 9e) [29]. Overall, planar MEGs serve as a fundamental platform for moisture-electric conversion, offering a simple architecture that can be scaled up to increase the total output and assembled easily with other electronics (Fig. 9f) [112].

### Fiber-Based Devices

**Fig. 10:**
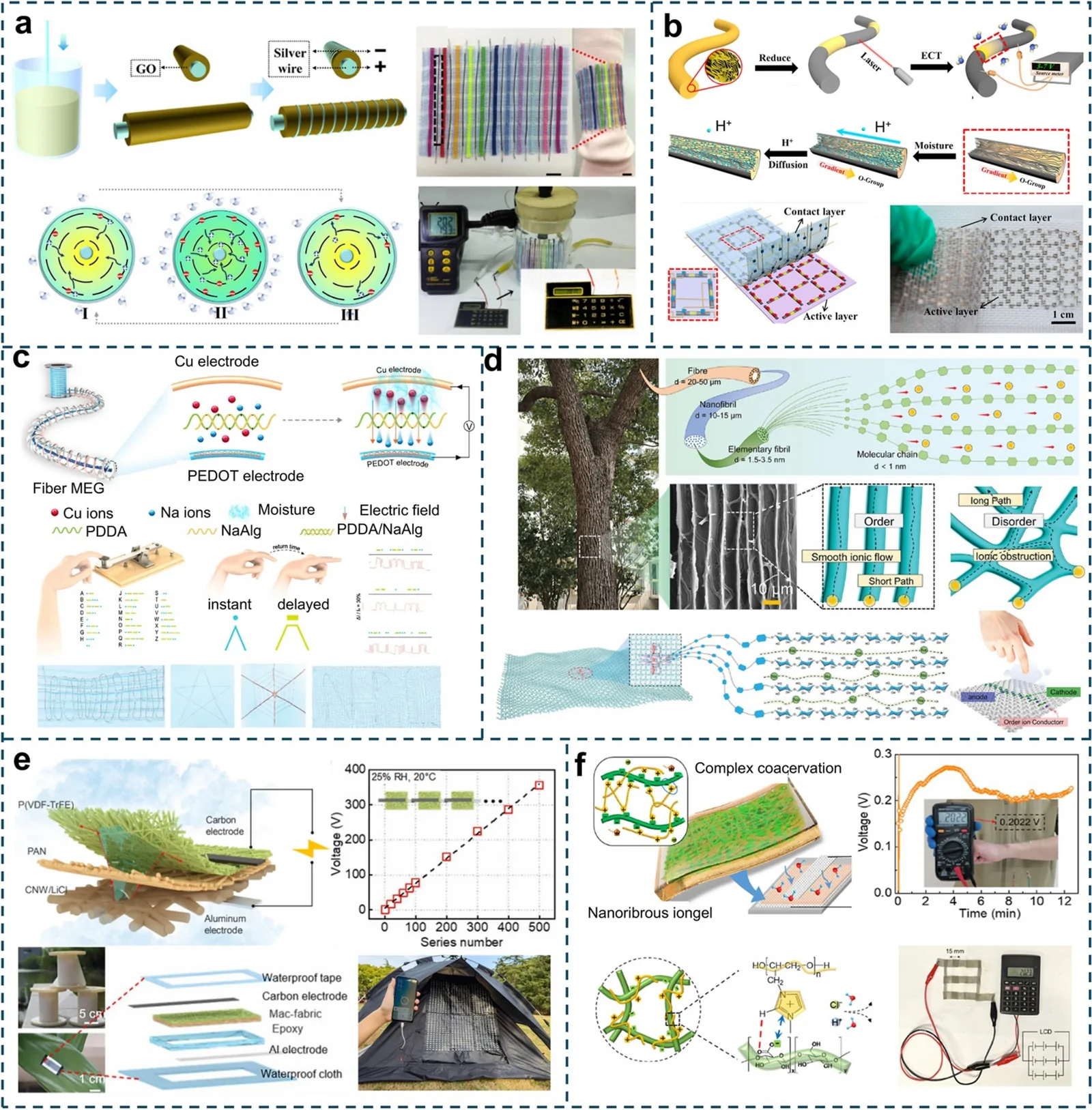
Fiber-based MEG devices. **a** Graphene oxide fiber for MEG. Reproduced with permission [142]. Copyright 2018, Elsevier. **b** Fiber MEG arrays fabricated by laser. Reproduced with permission [143]. Copyright 2017, Elsevier. **c** PEDOT filament MEG for information storage. Reproduced with permission [144]. Copyright 2024, Springer Nature. **d** Electrospun nano-fiber mats with asymmetric hygroscopic coatings for MEG. Reproduced with permission [145]. Copyright 2024, Wiley–VCH. **e** The fabric MEG with enhanced output. Reproduced with permission [146]. Copyright 2024, The American Association for the Advancement of Science. **f** Self-sustained moisture gradient and output with fabric in an open environment. Reproduced with permission [147]. Copyright 2024, American Chemical Society

As MEGs can provide energy source for other electronics including the wearable device, the flexibility of MEGs themselves is usually required. One strategy to improve flexibility and integration is to use materials such as fibers, yarns, and fabrics. In addition, fiber-based MEGs leverage the large surface-to-volume ratio of one-dimensional structures to adsorb water and generate ionic currents along the fiber length (Fig. 10a) [142]. A seminal example is the asymmetric graphene oxide fiber which were reduced by laser on selective regions to create conductive rGO sections adjacent to pristine GO sections (Fig. 10b) [143]. The rGO acts as an electrode and the GO as a proton-conducting electrolyte, establishing an internal gradient of functional groups. Upon a change in ambient humidity, a short rGO/GO junction fiber can produce ~ 0.4 V of potential difference [143]. This concept was also demonstrated by 1D polypyrrole nanowires doped with sulfonate showed that ion gradient along a fiber can drive electricity under moisture. Such single-fiber generators are highly flexible and can be woven or connected in series. Recently, a core–shell coaxial fiber design achieved a significant performance [144]. In this design, a conductive PEDOT filament core featured with a built-in immobile charge is coated with a hygroscopic polyelectrolyte gel shell made from a complex coacervate of PDDA and sodium alginate. The oppositely charged polymer pair in the shell provides abundant mobile ions, while the charged PEDOT core accelerates ion transport in the fiber. As a result, this uniaxial fiber MEG delivered up to ~ 0.8 V output with an impressive 1050 µA cm^−2^ current density even at RH of as low as 20% (Fig. 10c) [144]. Moreover, the PEDOT-core fiber showed exceptional mechanical robustness, enduring over 100,000 folding cycles with no performance loss.

Scaling from single fibers to textile generators, multiple fiber electrodes can be integrated into yarns and fabrics to create breathable, wearable power sources with moisture. In one approach, electrospun nano-fiber mats with asymmetric hygroscopic coatings are layered to form a Janus fabric that continuously harvests moisture from one side and releases it from the other [145]. Such designs can maintain a persistent moisture gradient within the textile, enabling continuous generation even after the hygroscopic region becomes saturated (Fig. 10d) [145]. The fabric-based MEG used a commercial polyester cloth coated partially with a hydrogel and carbon nanoparticle network. The wet region absorbed water from ambient air or sweat, while the other end of the fabric remained dry, creating an in-plane potential. Another study demonstrated a macrofiber fabric inspired by plant transpiration, achieving 14.4 µW cm^−2^ of power output at 40% RH by optimizing the yarn structure for fast moisture transport (Fig. 10e) [146]. As fabrics are inherently porous and permeable, they allow ambient air circulation, showing a potential as self-sustained moisture gradient in an open environment (Fig. 10f) [147]. Overall, fiber and textile MEGs combine energy harvesting with the advantages of fabric due to the lightweight, deformable characteristics. In addition, they can comfortably wrap around the human body or other surfaces, making them ideal for wearable and ubiquitous environmental powering applications.

### Multilayer and Stacked Configurations

**Fig. 11:**
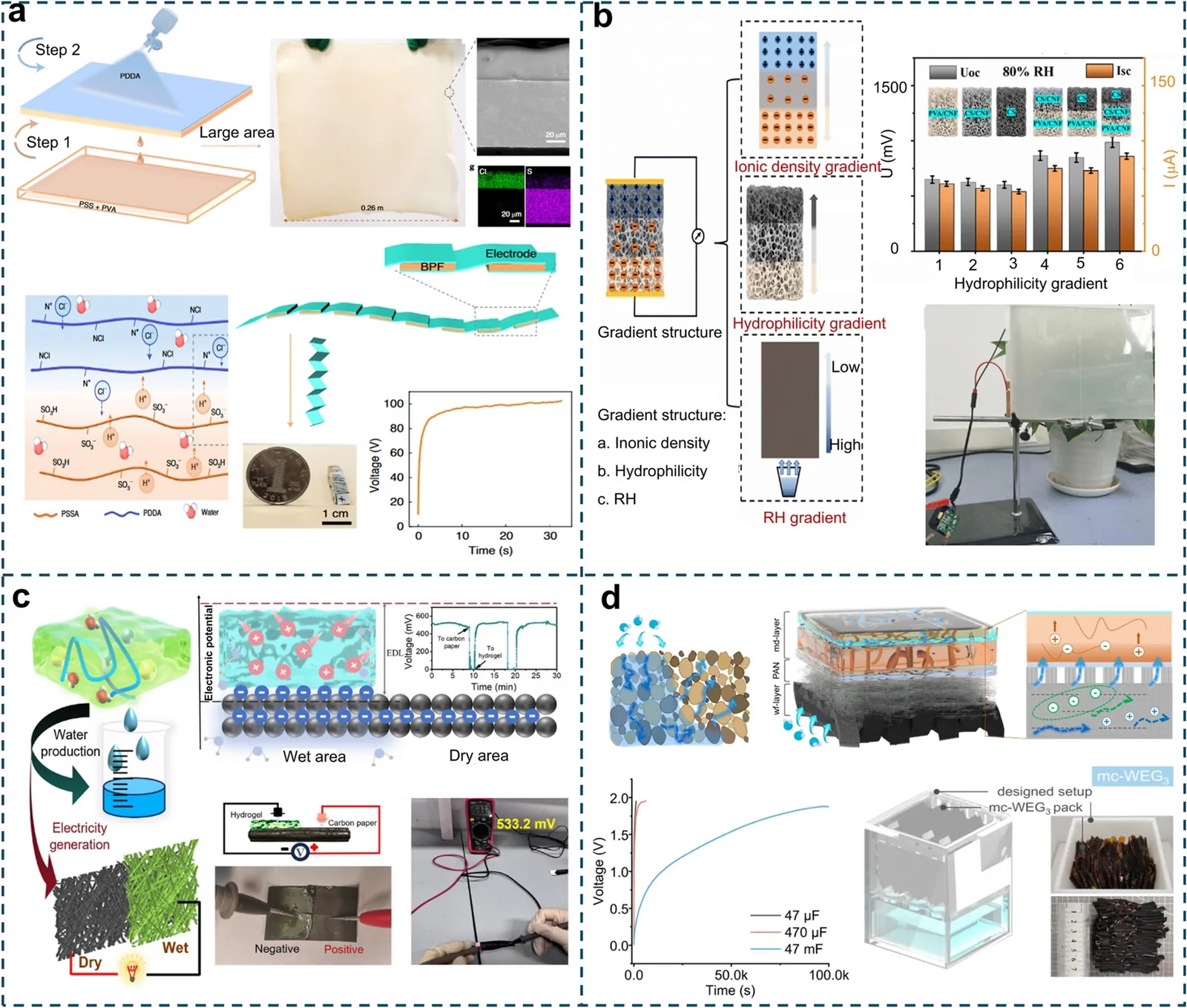
Multilayer and stacked configurations for MEGs. **a** Bilayer polyelectrolyte film device mimicking a transmembrane ionic gradient for MEG with enhanced electricity output. Reproduced with permission [15]. Copyright 2021, Springer Nature. **b** Double-gradient aerogel, combining a gradient in ion concentration with a gradient in hydrophilicity in the same porous matrix. Reproduced with permission [156]. Copyright 2023, Royal Society of Chemistry. **c** Incorporating multiple layers with wet and dry ends, exhibiting the possible of electricity generation in a self-sustainable manner. Reproduced with permission [157]. Copyright 2025, Wiley–VCH. **d** Multistage coupling water-enabled electric generator with customizable energy output. Reproduced with permission [158]. Copyright 2023, Springer Nature

To boost output voltage and power, multilayer architectures and stacking of MEG cells has been intensively investigated [15, 68, 73, 112, 148–155]. One strategy is to build composite layered generators that incorporate multiple functional layers in a single device [51–53]. A representative example is the bilayer polyelectrolyte film device, which mimics a transmembrane ionic gradient (Fig. 11a) [15]. This bilayer hybrid film consists of a polycationic PDDA film and PSSA/PVA film, which releases Cl^−^ ions and H^+^ ions when exposed to humid air, respectively. Thus, oppositely charged ions diffuses across the film, which largely improve the electricity output compared with sole film. As a result, such heterogeneous bilayer unit can generate ~ 0.95 V in ambient conditions. Stacking these internally asymmetric units in series yields a near-linear increase in total voltage. By connecting 1,600 bilayer units, an integrated device reached 1,000 V output under 25% RH. Stacked configurations can be in the form of 2D planar arrays or 3D multilayer stacks.

A double-gradient aerogel, combining a gradient in ion concentration with a gradient in hydrophilicity has the same porous matrix (Fig. 11b) [156]. The dual-gradient system facilitates continuous water transport and charge separation over long periods. As a result, the aerogel device achieved an industry-leading power density, which can operate steadily for ~ 120 h without performance loss. Such multilayer and hierarchical architectures address two major challenges: low single-cell voltage and short duration due to saturation. By incorporating multiple layers with different roles (e.g., one layer stays wet, another stays dry, or different ion types in each layer) (Fig. 11c) [157], these devices sustain a driving gradient. The trade-off is that complex multilayer fabrication must be well-controlled to avoid internal short-circuiting or high internal resistance. Researchers have used techniques like spray-casting, electrophoretic deposition, and freeze-drying to assemble such multi-layered structures. Overall, multilayer and stacked architectures represent a cost-effective and executable route to improve the output of MEG systems (Fig. 11d) [158]. Through clever material combinations and biomimetic gradients, these configurations achieve higher voltages and prolonged operation, which are crucial for real-world energy applications.

Although the overall output has been improved a lot through the scaling strategies, the electricity generated from MEG devices is still quite challenging for real-word applications. As the power density of single device unit is relatively low (below 100 µW cm^−2^) compared with other technologies such as solar cells, a great number of devices are needed to integrated, which increases the overall size as power supply. In addition, it is also challenging to keep great uniform output capability for each device, which bring the difficulties for device integration, especially the number of devices is huge. Furthermore, the output from MEG device is easily to be decayed with time, which is another great challenge for practical applications. To reduce the gap for real scenario, it is urgent to increase the power density and life-time of device output.

### Integration with Other Power Generation

The integration of MEG with other energy harvesting modalities represents an exciting frontier to bolster output performance and extend operational reliability. For instance, a recent innovative work has demonstrated a hybrid generator combining MEG with triboelectric energy harvesting driven by water droplets [14]. In this design, a polyglutamic acid–based hydrogel layer harvests electricity from evaporation and humidity, while a porous electret e-PTFE layer simultaneously generates triboelectric power through droplet impact. The hybrid device achieves DC output from MEG (~ 0.55 V, current density ~ 120 µA cm^−2^) and AC triboelectric output (~ 300 V, 400 µA), with sufficient power to drive wireless alarm and communication systems under harsh conditions such as cold or saltwater exposure. This example underscores how combining moisture-driven ionic currents with droplet-induced triboelectricity can dramatically amplify energy capture from natural water sources.

**Fig. 12:**
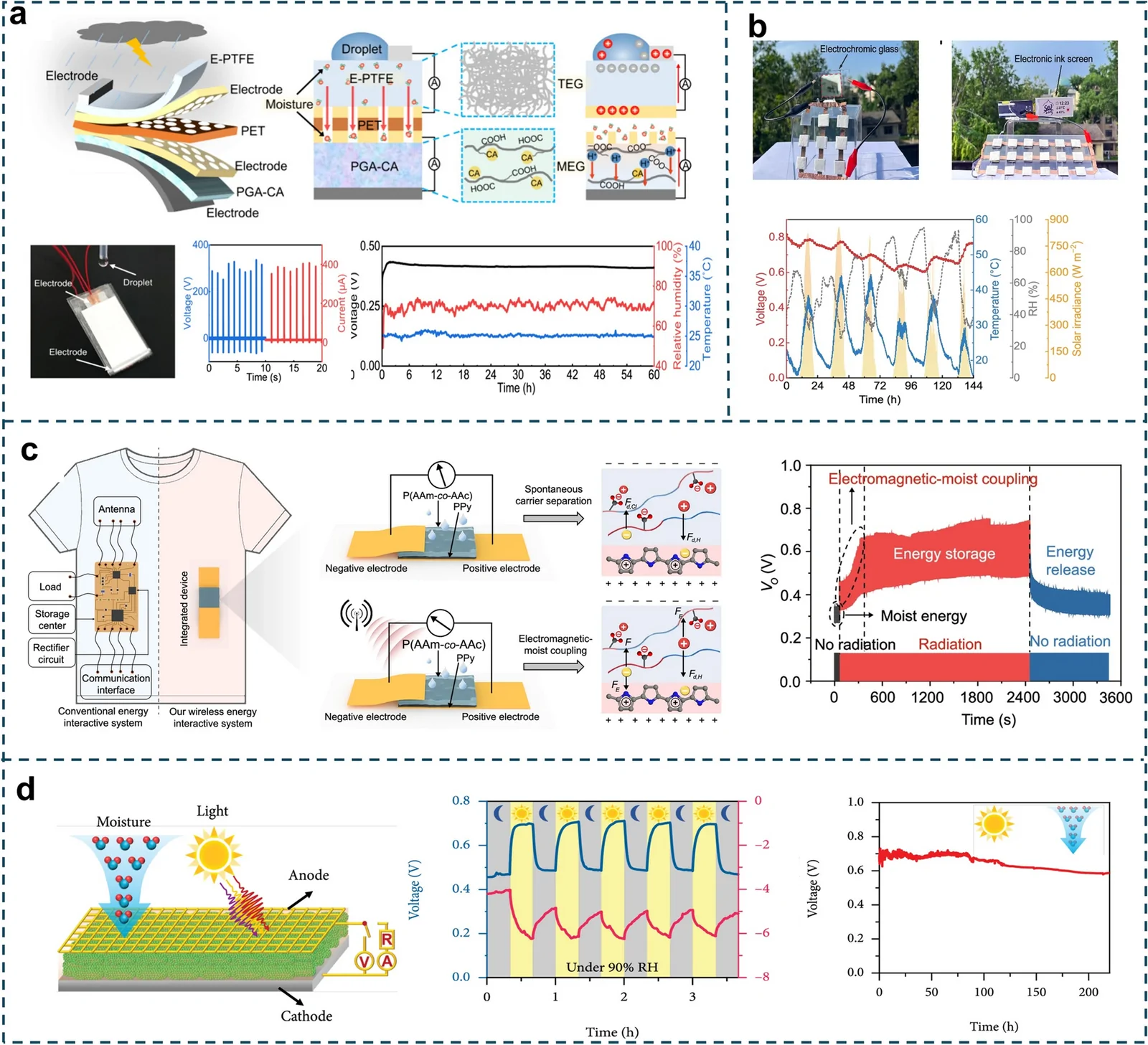
Integration of MEG with other power generation devices. **a** water triboelectrification complemented moisture electric generators for harvesting both moisture and tribo energies from water droplets. Reproduced with permission [14]. Copyright 2024, American Chemical Society. **b** Radiative cooling assisted self-sustaining and highly efficient moisture energy harvesting under cyclic sunlight. Reproduced with permission [61]. Copyright 2024, Springer Nature. **c** Wireless, chipless coupled interaction between electromagnetic and moisture energy interaction with the regulation of ionic diodes effect. Reproduced with permission [159]. Copyright 2025, Springer Nature. **d** All-biobased hydrovoltaic-photovoltaic electricity generators that integrate photosystem IIwith Geobacter sulfurreducens for simultaneous energy harvesting from both moisture and sunlight. Reproduced with permission [160]. Copyright 2022, American Association for the Advancement of Science

Another compelling hybrid approach involves integrating electromagnetic energy harvesting with moisture-electric generators via ionic diode films. Gao et al. developed a polyelectrolyte/conductive polymer ionic-diode film that exhibits diode-like moisture rectification [159]. This device couples atmospheric moisture harvesting with ambient electromagnetic wave absorption, enabling synergistic capture of both energy forms. Notably, the system achieves wireless, chipless hybrid energy harvesting—no separate antenna or rectifier is required—due to the ionic-diode effect modulating interfacial charge dynamics (Fig. 12). The design paves the way for flexible, integrated systems capable of simultaneous moisture and EM-driven power capture, offering niche applications in self-powered environmental sensing and wireless charging via humidity-based materials.

Beyond these recent innovations, hybridization concepts extend to combining MEG with photovoltaic elements, as demonstrated by hydro-photoelectric devices where moisture and solar illumination are concurrently harvested. A representative study reported an all-biobased hydrovoltaic–photovoltaic system that converts both ambient moisture and sunlight into electricity, offering a dual-mode energy capture without harsh materials—a compelling direction toward eco-friendly hybrid energy systems [160]. Further, theoretical frameworks have been proposed for evaporation-driven hydrovoltaic devices that harness thermodiffusion and photovoltaic effects in tandem, achieving open-circuit voltages up to 1 V and power density 25 µW cm^−2^ under combined heat and light stimuli. In addition to direct combining MEG with photovoltaic cells, the energy from sunlight can be used as thermal exchange with ionic hydrogel to establish a stable internal directed water/ion flow in MEG [61]. In this strategy, the radiative cooling of the top layer prevents the excessive daytime water evaporation under solar absorption while facilitating nighttime moisture sorption. As a result, the device can continuously generate a voltage of 0.88 V and current of 306 μA cm^−2^, demonstrating the operation outdoors for continuous 6 days.

Collectively, these hybrid configurations—moisture with triboelectric, electromagnetic, or solar energy generation—demonstrate how MEGs can be transformed from low-power, humidity-dependent devices into more robust and multifunctional energy harvesters. Each synergy leverages complementary mechanisms: triboelectric layers convert mechanical droplet impact, EM-coupled films absorb radiated energy, and OPV-like pairings tap into light or heat as well as ambient moisture. Such integrations address key limitations of MEGs, such as intermittent output or low power density, by broadening the temporal and modal energy capture spectrum.

## Applications

MEG has rapidly evolved from a laboratory curiosity into a promising power source for diverse applications in recent years. By harvesting ubiquitous ambient humidity or moisture from human or natural environments, MEG devices offer a green and broadly available energy supply that can complement traditional renewable sources. There are two primary approaches for applications of MEG devices: sensors and power sources. As the MEG output is sensitive to the external environmental conditions such as humidity and temperature, the device can be directly used as sensors for detecting these ambient physical parameters in a self-powered way. In addition, since moisture is existed everywhere, the MEG can generate electricity to power other electronics.

### Sensors

**Fig. 13:**
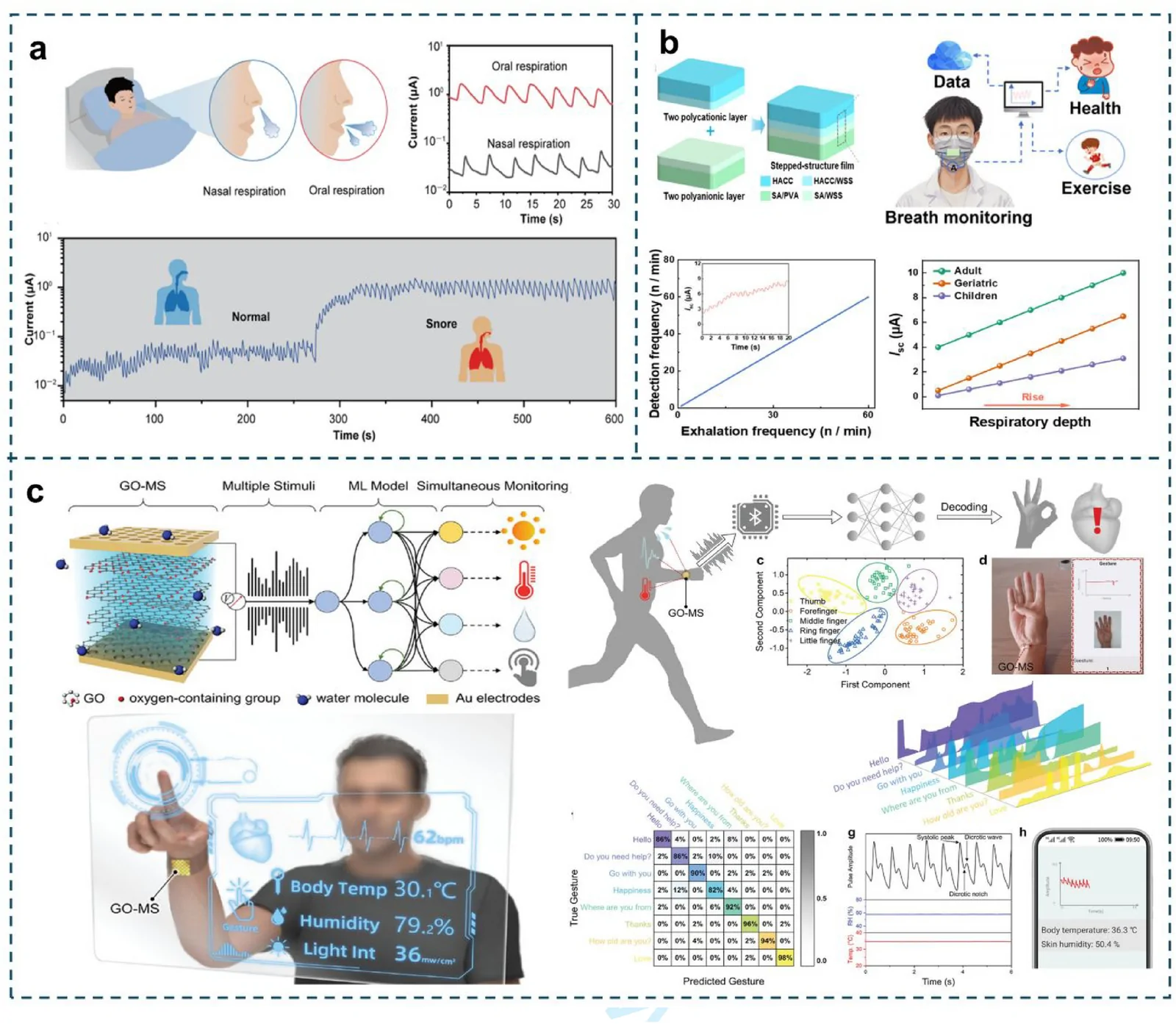
Applications of MEG as sensors. **a** MEG device as self-powered humidity sensor for night-time respiration monitoring. Reproduced with permission [161]. Copyright 2024, Springer. **b** MEG device as light and flexible breath monitor for detecting health conditions. Reproduced with permission [52]. Copyright 2025, Wiley–VCH. **c** A machine-learning-enhanced simultaneous and multimodal sensor based on MEG device from GO. Reproduced with permission [162]. Copyright 2022, Wiley–VCH

MEG-based power sources are inherently responsive to ambient humidity changes, making them natural candidates for self-powered environmental sensors. In fact, the electrical output of many MEG devices varies strongly with relative humidity, enabling dual functionality as both energy harvesters and hygrometers. For example, Ni et al*.* developed a tubular graphitic carbon nitride (g-CN) film on anodized aluminum oxide that acts as a MEG and humidity sensor simultaneously [161]. This device produced an open-circuit voltage of ~ 0.47 V and ~ 3.5 µA short-circuit current at 96% relative humidity (RH). More striking was its sensitivity: the output current changed by ~ 1.78 × 10^6^% when ambient RH increased from 41% to 96%, effectively translating tiny humidity fluctuations into large electrical signals. Exploiting this effect, the authors integrated the MEG sensor into a respiration monitor that could detect breathing patterns and even sleep apnea events in real-time, operating continuously for 12 h without any external power source (Fig. 13a). As MEGs is easily assembled into flexible, lightweight, and sensitive devices, they integration into wearable platforms such as commercial face masks for self-powered health monitoring, enabling real-time, 24/7 respiratory monitoring without external power (Fig. 13b) [52]. It has been demonstrated that the MEG devices can resolve respiration frequencies from 0–60 BPM, which covers the full range of medically relevant respiratory conditions. This demonstration highlights how MEG-based sensors can harness environmental moisture (in this case, exhaled breath) to both power themselves and transduce physiologically relevant humidity changes into electrical outputs for monitoring purposes.

Beyond personal respiration monitoring, MEG humidity sensors have been explored for broader environmental and multimode sensing. For example, the GO based MEG can generate electricity that is sensitive to both humidity and temperature (Fig. 13c) [162]. The output characteristic shows great linearity with the humidity from 3.8% to 26.7%, and the device has identical response signals in different humidity environments. In addition, the temperature can influence the mobility of the charges migration, hence affects the output as well. When the ambient temperature increases from 21 to 47 °C, the voltage of GO based MEG demonstrates a decline from 0.54 to 0.33 V. The device can also generate stable voltage signals and maintain a response pattern to even a slight temperature change of 0.3 °C, which demonstrate the great sensitivity with temperature. In addition, the porous structure of the GO based membrane is mechanically compressible. Under 1 N pressure, the output decayed rapidly from 0.54 to 0.41 V and gradually recovered to the initial level when the pressure was withdrawn. Thus, the device can be used for multimode sensing for humidity, temperature, and pressure, and the individual stimuli can be decoupled through machine-learning methods, making the simple MEG device as a smart sensor for self-powered human–machine interface.

### Power Sources

**Fig. 14:**
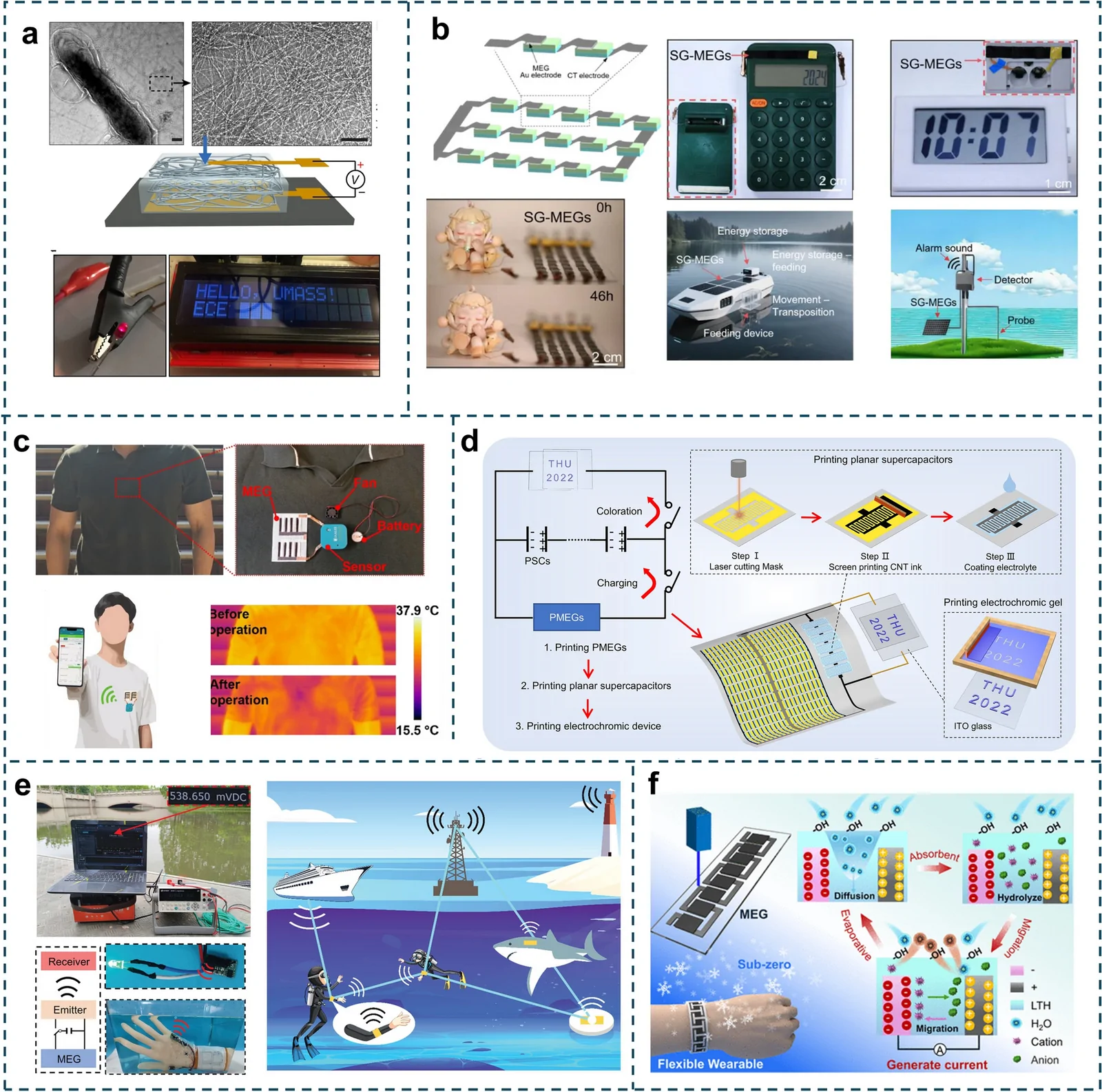
Application of MEG using as power sources. **a** Protein nanowire-based MEG for powering LED and LCD for display. Reproduced with permission [27]. Copyright 2020, Springer Nature. **b** An array of MEG for power electronic devices under various environments. Reproduced with permission [52]. Copyright 2025, Wiley–VCH. **c** A fan for cooling human body is powered by energy storage device charged by MEGs. Reproduced with permission [17]. Copyright 2025, Wiley–VCH. **d** Fully printed planar MEG arrays and supercapacitors for scalable function integration. Reproduced with permission [51]. Copyright 2023, Cell Press. **e** Demonstration of energy harvesting under water environment using MEG for powering marine electronics. Reproduced with permission [12]. Copyright 2024, Wiley–VCH. **f** Demonstration of electricity generation from MEG under low temperatures as power sources. Reproduced with permission [163]. Copyright 2025, American Chemical Society

Moisture-enabled electricity generation (MEG) devices have been tested as sustainable power sources for remote sensors in real or simulated field conditions. In one study, a thin-film MEG device made from networks of protein nanowires continuously harvested electric power from ambient humidity in the air. The device produced around 0.5 V, which is enough to power small electronics (Fig. 14a) [27]. By stacking multiple MEG units, the output voltage can be raised to a few volts, suitable for low-power circuits. For instance, a serial connection of 5 MEGs together increased the output voltage from 0.78 to 3.7 V, while the parallel integration of 10 MEG units produced an amplified current of 0.74 mA [52]. This modular scalability of MEGs highlights the capability and promise of MEG for real-world implementation, where usually customizable power output is often required. As a demonstration, a 5 × 8 serial-parallel of MEGs is sufficient to directly power 7 LED lights continuously for over 46 h. Furthermore, an array of three MEGs was adequate to power commercial devices such as digital calculators and LCD clocks. The MEGs could also be assembled into a self-powered wireless temperature and humidity monitoring system (Fig. 14b) [52]. The temperature and humidity sensor, signal processing and microcontroller units were driven by the power generated by an array of MEGs, where the temperature and humidity information can be sent to the smartphone through wireless communication system. The monitoring system can be used to any places where moisture exists, including agriculture, breeding industry, smart packaging, and human activity space, demonstrating great significance for the development of the Internet of Things.

The power generated from MEG units can be further used to charge electricity storage devices such as batteries and supercapacitors, where high power is needed. An electric fan connected to a Li-battery charged by MEGs can be attached onto the inner side of clothes for body cooling (Fig. 14c) [17]. After operation of cooling process for 1 min using this system, the human cloth area attached with electric fan can be cooling down by 22.70 °C. Developing a fully integrated self-powered system consisting of energy-harvesting, intermediate energy storage devices, and functional devices is promising for self-powered electronics. As MEG can be and flexible and printed into large area arrays, these power source can be integrated with printable supercapacitors to construct fully printed self-powered function system. Such system was demonstrated by MEG arrays for electricity generation, planar supercapacitors for intermediate energy storage, and ECD for information display (Fig. 14d) [51]. As a result, the patterned screen can be switched between the off and on states by controlling the voltage between the two electrodes using the power from MEG devices.

In addition to the power sources using in regular ambient environment, the MEG device can also be used for some extreme and specialized conditions such as underwater and freezing places, where the electricity power is challenging to be obtained. For example, harvesting energy under liquid water based on MEG technology was first demonstrated by Shen’s group [12]. The operationality of MEG from air to underwater environment was achieved through a smart design, where the sandwiched engineered-hydrogel device was covered with an additional waterproof breathable membrane layer made from porous E-PTFE film allowing water vapor exchange while preventing liquid water penetration (Fig. 14e) [12]. Impressively, under liquid water environment, the device can deliver a voltage of 0.55 V and a current density of 130 µA cm^−2^. In addition, the output can be maintained even under harsh underwater environment with 10% salt concentration, 1 m s^−1^ disturbing flow, as well as > 40 kPa hydraulic pressure. Even under the high-concentration salt solutions, the device can produce a remarkable output, which holds a great promise in the creation of a new range of innovative electronic devices for marine Internet-of-Things. In addition, by reducing hydrogen bonds between water molecules, a low-temperature-resistant low-temperature hydrogel can be used for electricity generation under icing temperature [163]. At − 35 °C and RH = 16%, the MEG unit can produce voltages up to ∼0.58 V and currents up to ∼14.35 μA, which can be used to drive electronics such as watches under extremely cold environment.

## Challenges and Future Directions

Over the past decade, the MEG technology has developed intensively. However, there are still some challenges in this field. Competing hypotheses and a lack of unified theory hinder rational design, a more in-depth, molecular-level understanding is needed to predict and optimize MEG performance. Many MEG devices exhibit performance degradation over time, with output dwindling as hygroscopic materials dry out or electrodes foul. Recent advances have extended continuous operation into the multi-day range, but maintaining stable performance over long durations remains a core challenge. Typical devices yield on the order of millivolts and microampere currents, translating to very small power densities. Such output is often too low and inconsistent to directly drive electronic devices. Furthermore, current MEG designs may require bulky hygroscopic layers or liquid reservoirs ill-suited for compact electronics. Integrating MEGs into wearables or IoT sensors has proven challenging due to their low output and lack of flexibility or breathability. Equally important is the electrical integration: MEG outputs (often DC with high internal resistance) need power management (boost converters, regulators) to interface with conventional electronics, adding another layer of complexity. To date, large-scale integration of MEGs has not been achieved in practice. Overcoming these issues will involve developing manufacturing techniques for consistent large-area hygroscopic films and modular device architectures. Thus, challenges still remain in realizing high output from scaled-up or arrayed devices. Printing techniques and roll-to-roll fabrication of MEG materials are potential pathways to improve scalability. Despite that many challenges exist for the practical applications of MEGs currently, there are still many opportunities remaining in this field. We summarize the future directions as follows:

1. **Emerging materials for improved performance.** A key frontier is the development of new material systems that can yield higher outputs and greater durability. Metal–organic frameworks, with their high porosity and tunable surface chemistry, are being explored to enhance moisture uptake and charge separation. Supramolecular and nanocomposite materials are also promising. Recent work with a polyvinyl alcohol/sodium alginate supramolecular hydrogel achieved short-circuit current densities in the milliamp range, far outperforming earlier devices. Additionally, biodegradable or bio-derived materials hold great promise to create eco-friendly MEGs. Using natural materials such as polymers can reduce toxicity and even allow the device to self-degrade or be recycled at end-of-life. The overarching trend is toward materials that are highly hygroscopic, ion-conductive, and environmentally benign, which will lead to MEGs with better performance and broader application potential.
2. **Hybrid energy-harvesting techniques.** To overcome the limitations of a single-source harvester, future MEG implementations will likely be part of hybrid generators combining multiple energy modalities. By coupling moisture-electric generation with solar, thermal, or mechanical energy harvesters, one can take advantage of different energy sources throughout the day or under varying conditions. For instance, combining a moisture-adsorption cycle with a solar-driven thermal cycle has enabled 24 h power generation. During day time, solar heat assists water desorption and drives a thermoelectric generator, while at night radiative cooling and moisture reabsorption continue to produce power. Similarly, electromagnetic–moisture hybrids are also promising, where an ionic diode film harvests ambient electromagnetic energy in tandem with moisture-induced ionic currents. Looking ahead, we can envision MEGs integrated with triboelectric nanogenerators or with solar cells, creating multi-source energy packs.
3. **Self-regulating MEGs and energy storage integration.** Another future direction is making MEG systems smarter and more self-sufficient. Self-regulating MEGs would be able to adapt to environmental changes by adjusting their operation or internal configuration. Future MEGs might incorporate microfluidic control or stimuli-responsive materials to modulate moisture levels and maintain optimal performance automatically. In tandem with self-regulation, integrating energy storage is crucial for practical use. This means pairing MEGs with small batteries or supercapacitors to smooth out intermittent output and store excess energy. There are early demonstrations where the electricity from a moisture generator is stored in a capacitor and later used to power an electronic device once sufficient charge is accumulated. In future, compact integrated units could include a moisture-harvesting component and a solid-state micro-battery in one package. Such integration of harvesting and storage will make the energy supply far more reliable and enable MEG-powered systems to function autonomously through environmental fluctuations.
4. **AI-assisted material and device optimization.** The application of artificial intelligence and machine learning is poised to accelerate advances in MEG technology. AI can help navigate the vast design space of materials and configurations much faster than traditional trial-and-error experimentation. AI accelerates the identification of candidates with high moisture-to-electric conversion potential by predicting key properties (water uptake, ionic conductivity, etc.) before synthesis [164]. This data-driven foresight steers researchers toward the most promising materials, replacing exhaustive trial-and-error with targeted exploration. For example, machine learning models can rapidly screen databases of polymers or MOFs to find those that absorb moisture abundantly and conduct ions effectively [165]. AI also aids in optimizing material structure and composition: a notable case used transfer learning to refine a nanochannel design, boosting output voltage of a MEG device [166]. By uncovering complex structure–property relationships, AI not only quickens discovery but also inspires novel material designs (e.g., gradient films, new composites) for moisture-electric generators. Such AI-driven optimization can promote accurate performance prediction and multi-parameter optimization, yielding designs that might have been overlooked by intuition. Moving forward, we anticipate increasing use of data-driven methods to discover new hygroscopic material formulations, predict long-term stability, and identify the best device architectures under various climatic conditions. AI could also assist in real-time control, where smart MEG systems learn to adjust themselves for maximal efficiency. Incorporating AI and machine learning into the device development will likely hasten the arrival of high-performance and robust MEGs.
5. **Real-world application development.** Finally, the true test for MEG technology will be the performance in real-world scenarios. Future efforts will focus on fields that integrate MEGs into practical devices. Encouragingly, we are starting to see early deployments that recent prototypes have successfully powered a digital clock continuously for a month, illuminated LED bulbs, and even charged a smartwatch, all using ambient humidity as the energy source. In the realm of wearables, moisture-driven fabric devices can be worn and produce electricity from the user’s perspiration or ambient air, aimed at self-powered health sensors. Environmental sensor nodes driven by MEGs are also being explored for deployment in remote, humid environments where replacing batteries is inconvenient. In biomedical devices, the goal is to harvest moisture from sweat to power biometric sensors, eliminating the need for bulky batteries. The coming years are likely to see a transition from proof-of-concept experiments to integrated MEG systems operating in real-world conditions.
